# Press touch code: A finger press based screen size independent authentication scheme for smart devices

**DOI:** 10.1371/journal.pone.0186940

**Published:** 2017-10-30

**Authors:** M. S. A. Noman Ranak, Saiful Azad, Nur Nadiah Hanim Binti Mohd Nor, Kamal Z. Zamli

**Affiliations:** 1 Faculty of Computer Systems and Software Engineering, University Malaysia Pahang, Gambang, Kuantan, Malaysia; 2 IBM Center of Excellence, UMP, Gambang, Kuantan, Malaysia; King Saud University, SAUDI ARABIA

## Abstract

Due to recent advancements and appealing applications, the purchase rate of smart devices is increasing at a higher rate. Parallely, the security related threats and attacks are also increasing at a greater ratio on these devices. As a result, a considerable number of attacks have been noted in the recent past. To resist these attacks, many password-based authentication schemes are proposed. However, most of these schemes are not screen size independent; whereas, smart devices come in different sizes. Specifically, they are not suitable for miniature smart devices due to the small screen size and/or lack of full sized keyboards. In this paper, we propose a new screen size independent password-based authentication scheme, which also offers an affordable defense against shoulder surfing, brute force, and smudge attacks. In the proposed scheme, the Press Touch (PT)—a.k.a., Force Touch in Apple’s MacBook, Apple Watch, ZTE’s Axon 7 phone; 3D Touch in iPhone 6 and 7; and so on—is transformed into a new type of code, named Press Touch Code (PTC). We design and implement three variants of it, namely mono-PTC, multi-PTC, and multi-PTC with Grid, on the Android Operating System. An in-lab experiment and a comprehensive survey have been conducted on 105 participants to demonstrate the effectiveness of the proposed scheme.

## Introduction

Recent enhancements to smart devices and their appealing applications make them desirable to consumers of all ages. Hence, consumers around the globe are embracing smart devices at a greater ratio. In 2014, around 1.75 billion users worldwide own and use smartphones, which are 25% higher than the earlier year [1]. At present, smart devices are considered as the modern-day’s constant companions of human beings. For that reason, people store several private information—such as contact details, essential documents, secret and public images, PIN numbers, and other valuable data—in their devices for frequent access. Again, these data turn these devices vulnerable to various attacks since the primary reason of attacking these devices is to acquire data. In the recent past, a considerable number of attacks have been noticed [2]. Therefore, ensuring security of these devices become a burning issue; and hence, many smart devices employ one or more authentication schemes.

Among various authentication schemes, password-based authentication schemes are the most common type of schemes that are utilized on many smart devices due to their lower implementation complexities, lower computational complexities, lower processing requirements, and so forth. Again, text-based authentication schemes are more common than other existing password-based schemes [3, 4]. However, several cryptanalysts discovered various vulnerabilities in text-based schemes, e.g., dictionary attack [5], social engineering attack [6], brute force attack [7], guessing attack [8], etc. Moreover, the tiny screen size of the smart devices imposes some more constraints in text-based schemes, e.g., limited length password and small on-screen keyboard. Due to the latter constraint, typing turn out to be less precise and inefficient. Consequently, people use even smaller passwords, which make them additionally vulnerable. Again, in many miniature smart devices—such as smart watch, smart band, and so forth—this type of passwords are not suitable due to unavailability of full/partial keyboards. Hence, most of them are not screen size independent.

For smart devices, graphical password schemes are preferred due to several reasons, such as *i*) these schemes are heavily graphic oriented in nature, *ii*) memorability of these schemes are higher over text-based schemes—in several psychological studies, it has been identified that humans can remember images more than their counterparts, *iii*) these schemes offer a larger password space compare to text-based schemes, and so on. However, graphical password schemes are vulnerable to several attacks, e.g., shoulder surfing [9], smudge attack [10], intersection attack [11], reflection attack [12], and so on. Moreover, they also experience some serious problems, such as fat finger problem [13], tiny image problem, and so forth. In fat finger problem, a user has difficulty in using a touchscreen device because the fields or buttons of the applications are too small for the width of the finger. Android pattern lock [14], tiny lock [15], pass-go [16], and other resembling schemes suffer from this. It is even prominent in miniature smart devices for their limited screen sizes. On the other hand, the tiny image problem is more common among the image selection based graphical password schemes [17–20]. It is eminent in the devices with limited screen size. Therefore, alike text-based schemes, most of the graphical password schemes are also not suitable for the miniature smart devices. In other words, most of them are not screen size independent.

Although, smart devices come in different sizes—most of the existing password-based authentication schemes are not screen size independent as argued in earlier discussions. Therefore, they fail to ensure the security of all sized smart devices. Hence, it remains an important issue to investigate. In this paper, we tackle this issue by proposing a new screen size password-based independent authentication scheme, which transforms the existing Press Touch (PT) into a new type of code, named Press Touch Code (PTC). This code can be applied on any smart devices irrespective of their sizes. We propose three variants of the PTC, namely mono-PTC, multi-PTC, and multi-PTC with Grid. These variants offer different level of security. All these three variants are implemented on the Android Operating System; and they are tested using a Huawei P9 Plus device, which is a pressure sensitive technique enabled device. More details of the proposed scheme and analysis of it are mentioned in the Proposed scheme and Analysis of our proposed scheme Sections, respectively.

Our contributions in this work could be summarized as follows:

- A novel screen size independent authentication scheme is proposed that utilizes the press touch technique of smart devices and offers an affordable defense against shoulder surfing, brute force, and smudge attacks.
- A technique of transforming the press touch into a new type of code—called press touch code—is developed, which is utilized as credentials for authenticating individuals.
- Three variants of the proposed scheme are designed and implemented on the Android Operating System, which offer different level of security.

The subsequent sections of the paper are organized as follows. The next section presents all the relevant screen size independent authentication schemes and their limitations. In the subsequent section, our proposed scheme is detailed with a relevant algorithm. Afterwards, the security and usability evaluations of the proposed technique are discussed. Then, the validity threats associated with the in-lab experiment and the survey are elaborated and debated. This paper ends with the concluding remarks.

## Related works

To protect smart devices from various threats and attacks, most of the devices employ one or multiple authentication schemes. These schemes could be broadly classified as: *i*) password-based schemes, *ii*) biometric schemes, and *iii*) hybrid schemes. As argued in the Introduction Section, most of the password-based schemes—which mainly includes text-based schemes [21–23] and graphical schemes [14–20], [24], [25], [26]—are not screen size independent.

Among the existing techniques, most comparable to our proposed scheme—which are fully or partially screen size independent—are knock code [25] and vibration code [26]. Again, the knock code of the recent LG devices are not directly implementable on all devices. For miniature devices, the existing—2 × 2 grid—has to be reduced to 1 cell for the adaptation. One of the major limitations of this scheme is that it is vulnerable to shoulder surfing attack—where an attacker can attain the password of a user by picking on the screen or by capturing the video of the entire authentication session. On the other hand, our proposed scheme offers an affordable defense against the shoulder surfing attack. To prove that an in-lab experiment has been performed taking both the schemes into account and the results are demonstrated in the Evaluation Section.

On the other hand, the Vibration Code (VC)—which is an integral part of Vibration And Pattern (VAP) code—is a screen size independent authentication scheme. Although, VC is a screen size independent scheme, but VAP is not. The VC utilizes the vibrations of the existing smart devices and transformed them into a code, and hence the name. Since it is a sense based technique, it can resist shoulder surfing attack, and other two prominent attacks, e.g., smudge and brute force attacks. However, this scheme spends a considerably longer duration for authentication. For instance, for a total VC of *β*_*t*_ (where $\beta_{t}\in Z^{+}$), the authentication duration lies between (*β*_*t*_ × *τ*_*min*_ + |*Q*| × *τ*_*g*_) to (*β*_*t*_ × *τ*_*max*_ + |*Q*| × *τ*_*g*_), where *τ*_*g*_ is the average interval to move from one grid to another grid. If *τ*_*min*_ = 300*ms*, *τ*_*max*_ = 900*ms*, |*Q*| = 4, *β*_*t*_ = *sum*(*S*) = 10, and *τ*_*g*_ = 250*ms*, then the duration of authentication lies between 4 seconds to 10 seconds [26]. With compare to other existing schemes, it is a considerably large duration. This scheme is preferable where security is imperative and there is limited or no timing constraint. Generally, most of the smart device users prefer those schemes, which offer shortest authentication duration. Consequently, VC is not preferred by many smart device users.

On the other hand, there are some smart devices, which employ biometric authentication schemes [27–31]. Although, many of these schemes are screen size independent; however, for enabling these schemes require special hardware and a considerable amount of computational power. Hence, they are not suitable for miniature smart devices and found only on high-end smart devices. Similar arguments are also applicable for hybrid schemes since they combine password-based and biometric-based approaches together. Therefore, an alternative is obligatory, and in this paper, we propose a such scheme, which is elaborated in the subsequent section.

## Proposed scheme

In this paper, we propose a new authentication scheme, which exploits the existing Press Touch (PT) technique of various smart devices by transforming it to a new type of code, named Press Touch Code (PTC). Generally, PT is utilized to produce haptic feedback and to elicit a different set of responses depending on the intensity of the pressure applied on the touchscreen. This technique is also known as Force Touch in Apple’s MacBook, Apple Watch, ZTE’s Axon 7 phone; 3D Touch in iPhone 6 and 7; and so on. In this paper, we adopt the term—Press Touch—since Huawei [32] has given this name to their Pressure Sensitive Technique (PST) and our proposed scheme has been implemented and tested on one of the Huawei devices; more specifically, Huawei P9 Plus. This scheme is also directly applicable to other PST enabled Android devices. It also could be enabled to other similar devices of different operating systems with necessary modifications.

Note that the pressure sensitivity technique is never been utilized as an authentication scheme; and hence, PTC is the first of its kind. However, it has a lot of potentials, which we exploit in this work. The PTC could be utilized as a stand-alone authentication scheme or for higher security, it could be extended with multiple similar codes or again the latter also could be enhanced by incorporating grid cells in it. Let us distinguish them by calling mono-PTC, multi-PTC, and multi-PTC with Grid, respectively. Since the latter two variants are the extended version of the former variant; therefore, for grasping the idea of these variants, the detail knowledge about the former one is mandatory. Consequently, our subsequent discussions are arranged accordingly.

### Mono-PTC

When a user places a finger on a screen, the Pressure Sensitive Screen (PSS) can recognize the intensity of the touch. Let us denote this as $\zeta_{t_{0}}$, which is the intensity of the press at time *t*_0_. Afterwards, the intensity of the press is measured after every Δ*t* time unit. This measured value ranges between 0 to 1, where 1 is for the most intense press and 0 is for no touch on the screen. For that reason, the press intensity value of a PT shows direction towards 1. In our proposed scheme, we utilize these values to generate a code, which would be considered as a password or signature of a particular user. In this paper, password and signature terms are used interchangeably.

The entire process of placing a finger on the screen to proving PTs to generating PTCs could be divided into three phases, namely *i*) data acquisition, *ii*) data cleaning, and *iii*) press touch finding. All these phases are discussed below in details.

**Fig 1:**
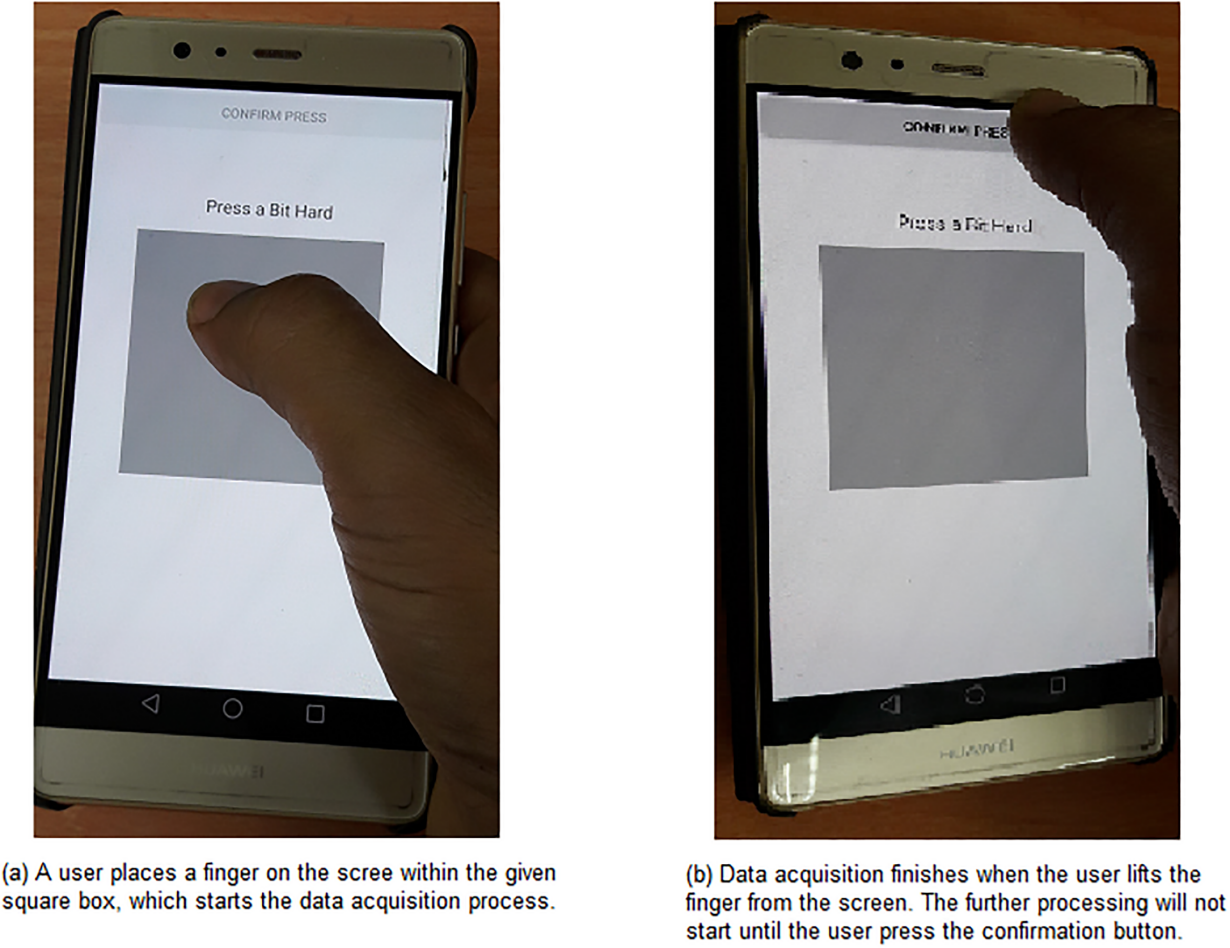
Data acquisition process.

**Fig 2:**
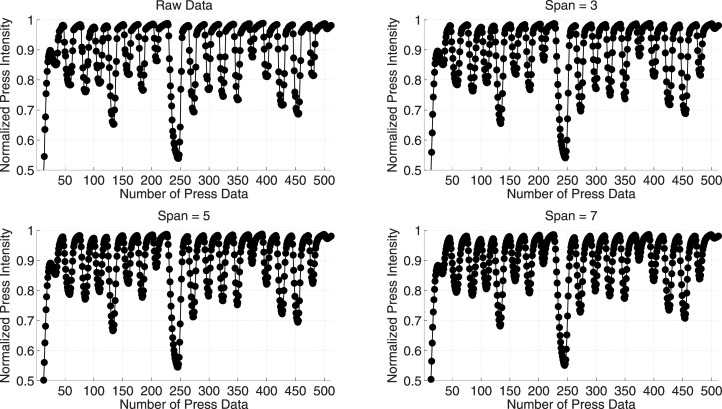
The acquired data and the smoothed data for various spans are shown.

**Data acquisition:** The data acquisition phase starts when a user places a finger within the given box on the screen as shown in Fig 1. Although, data could be acquired from any place on the screen, but the box is given to expel any confusion that may arise in deciding where to press. Let us assume that $\zeta_{t_{0}}$ is the first press intensity value acquired at time *t*_0_. Afterwards, the data acquisition process keeps acquiring press intensity values after every Δ*t* time unit and stores them in a vector, *ζ*, where $\zeta =\{\zeta_{t_{0}},\zeta_{t_{1}},...,\zeta_{t_{n}}\}$, $0\leq \zeta_{t_{i}}\leq 1$, and *t*_*i*+1_ − *t*_*i*_ = Δ*t* where *i* = 1, 2, 3, …, *n*. During this process, a user provides a desire PTC by pressing forcefully—which is a.k.a. PT—for that number of times. For instance, if a user has decided a *PTC* of *k*, where $k\in Z^{+}$, then s/he must provide *k* PTs or in other words, press forcefully for *k* number of times. The whole procedure must be quick and sharp; otherwise, noise will be introduced due to finger movement as fingers seldom remain steady [33]. To end the data acquisition procedure, a user must lift the finger from the screen. Once all the data are acquired (a sample data set is provided in S1 File), they are cleaned and processed later to extract the PTC. The subsequent phases will not begin until the user presses the confirmation button as shown in Fig 1b.
**Data cleaning:** In this phase, the acquired data are cleaned to remove unwanted noises from them. For extracting exact PTC, this phase is immensely important since any noise in the data can hamper the calculation. Moreover, a clean data simplify further processing.
  In our proposed technique, we employ Moving Average Filtering Technique (MAFT) [34] for cleaning the acquired data. In MAFT, the data are smoothed by replacing each data point with the average of the neighboring data points defined within a span. For this, following equation (i.e., Eq (1)) has been applied on *ζ*:
  $$\begin{array}{c} \zeta_{t_{i}}^{\prime }=\frac{1}{2N+1}(\zeta_{\left(t_{i}+N\right)}+\zeta_{\left(t_{i}+N-1\right)}+\ldots +\zeta_{\left(t_{i}-N\right)}) \end{array}$$ (1)
  where $\zeta_{t_{i}}^{\prime }$ is the press intensity value after smoothing process for the *i*th data point, *N* is the number of neighboring data points on either side of $\zeta_{t_{i}}$, and 2*N* + 1 is the span. In Fig 2, an example of smoothing operation is shown for three *N* values, namely 1, 2, and 3; and thus, three span values, namely 3, 5, and 7, respectively. Although, *span* = 5 and 7, clean the data more than *span* = 3; however, in some cases, they flatten the peak and impose complexity in finding the code. Therefore, in our proposed scheme, we adopted *span* = 3, which smoothes the data considerably enough for finding the exact code by employing a simple algorithm. Afterwards, $\zeta_{t_{i}}\leftarrow \zeta_{t_{i}}^{\prime }$, and $\zeta_{t_{i}}^{\prime }$ is erased.
**Press touch finding:** As it could be observed from the Fig 2 is that when a user provides a PT—a peak is generated. Therefore, in the rest of the discussion, these two terms are utilized interchangeably. Any suitable 1-D Peak finding algorithm [35] could be utilized as Press Touch Finding Algorithm (PTFA) with some modifications. One notable modification is that instead of finding a global optimum peak, the PTFA always has to discover local maximum peaks. Moreover, it also has to count the number of peaks since it is the PTC given by the user during the authentication session.
  In our proposed scheme, a brute-force technique based PTFA is employed to discover all the local maximum peaks since the number of elements in *ζ* is considerably lower. Other factor that influence us in selecting brute-force technique is that it is simple to implement and requires comparable computational power when number of elements are lower like our scenario. Time complexity of this algorithm is O(n). In this algorithm, any press intensity value, $\zeta_{t_{i}}$ is a peak if it is greater than its neighbor(s), i.e.,
  $$\begin{array}{c} \zeta_{t_{i}-1}<\zeta_{t_{i}}>\zeta_{t_{i}+1} \end{array}$$ (2)
  where $\zeta_{t_{0}}=\zeta_{t_{n}+1}=-\infty$. Eq (2) is applied on *ζ* to discover all the local maximum peaks and as mentioned before, number of local maximum peaks are equivalent to number of PTs. For instance, in Fig 2, there are 20 local maximum peaks, which means that there are 20 PTs, i.e., *PTC* = 20. This value is then either store (in case of registration) as a signature of the particular user or compare (in case of authentication) with the registered signature to match the similarity. The pseudocode of this algorithm is detailed in Algorithm 1.

**Algorithm 1** Press touch / Peak finding algorithm

1:  *LocalPeakFound* ← *false*;

2:  *TotalPeakFound* ← 0;

3:  *Data*[−1] ← −∞;

4:  *Data*[*n*] ← −∞;

5:  **for** *i* ← 0 to *Data*.*size* − 1 **do**

6:   **if** *Data*[*i* − 1] ≤ *Data*[*i*] && *Data*[*i*] ≥ *Data*[*i* + 1] **then**

7:    *LocalPeakFound* ← *true*;

8:   **else**

9:    *LocalPeakFound* ← *false*;

10:   **end if**

11:   **if** *LocalPeakFound* = *true* **then**

12:    *TotalPeakFound* + +;

13:    *LocalPeakFound* ← *False*;

14:   **end if**

15: **end for**

    **return** *TotalPeakFound*;

### Multi-PTC

Although, it is possible to have a considerably large PTC; however, during our survey—detailed in the Analysis of the proposed scheme Section—we observed that participants usually prefer moderate PTC values. Let us consider that the highest PTC provided by any user is *μ*. Hence, this is the highest achievable password space using mono-PTC. Such a small password space is not adequate to defend the brute force attack. However, password space can be enlarged by repeating the mono-PTC for several cycles. Thereby, the mono-PTC is extended with multiple cycles, named multi-PTC.

In multi-PTC, a user has to repeat mono-PTC for multiple cycles with an interval constraint. S/he has to repeat subsequent mono-PTC within a fixed time interval, *τ*. Again, *τ* must be cautiously selected since a large value of *τ* would result in a long authentication session and a smaller *τ* would result in expiration before starting the subsequent cycle. Both these *τ* values would reduce the usability of the proposed scheme. Long story short, a *τ* must be chosen reasonably small considering the usability issues of the user.

After completing one cycle of PTC, a user must start the subsequent cycle within the *τ* time unit. Conversely, it would be considered as the end of the authentication session. Since PTs are acquired in various cycles, a 2 − *D* data structure, $\zeta_{t_{ij}}$ is employed to store press intensity values; where *i* is the index of a 1 − *D* data structure, e.g., a vector like in mono-PTC, *j* is the index of the cycle, and $i,j\in Z^{+}$. At the end of the data acquisition session, alike mono-PTC, all the data are cleaned using MAFT with *span* = 3. Afterwards, PTFA is employed on every *i* to discover corresponding PTC, denoted as *ρ*_*i*_. Every *ρ*_*i*_ is then stored in a vector, *S*; where *S* = {*ρ*_0_, *ρ*_1_, …, *ρ*_*m*_} and *m* is the number of PTC giving cycles. In other words, *S* is the signature of the user, which s/he has to repeat during the authentication session. The password space of this scheme can be calculated as *μ*^*m*^. For instance, when *μ* = 10 and *m* = 4, it offers a password space equivalent to a 4-digit PIN, i.e., 10^4^. That means, multi-PTC has affordable resilience against the brute force attack and it outperforms mono-PTC in this respect.

### Multi-PTC with grid

**Fig 3:**
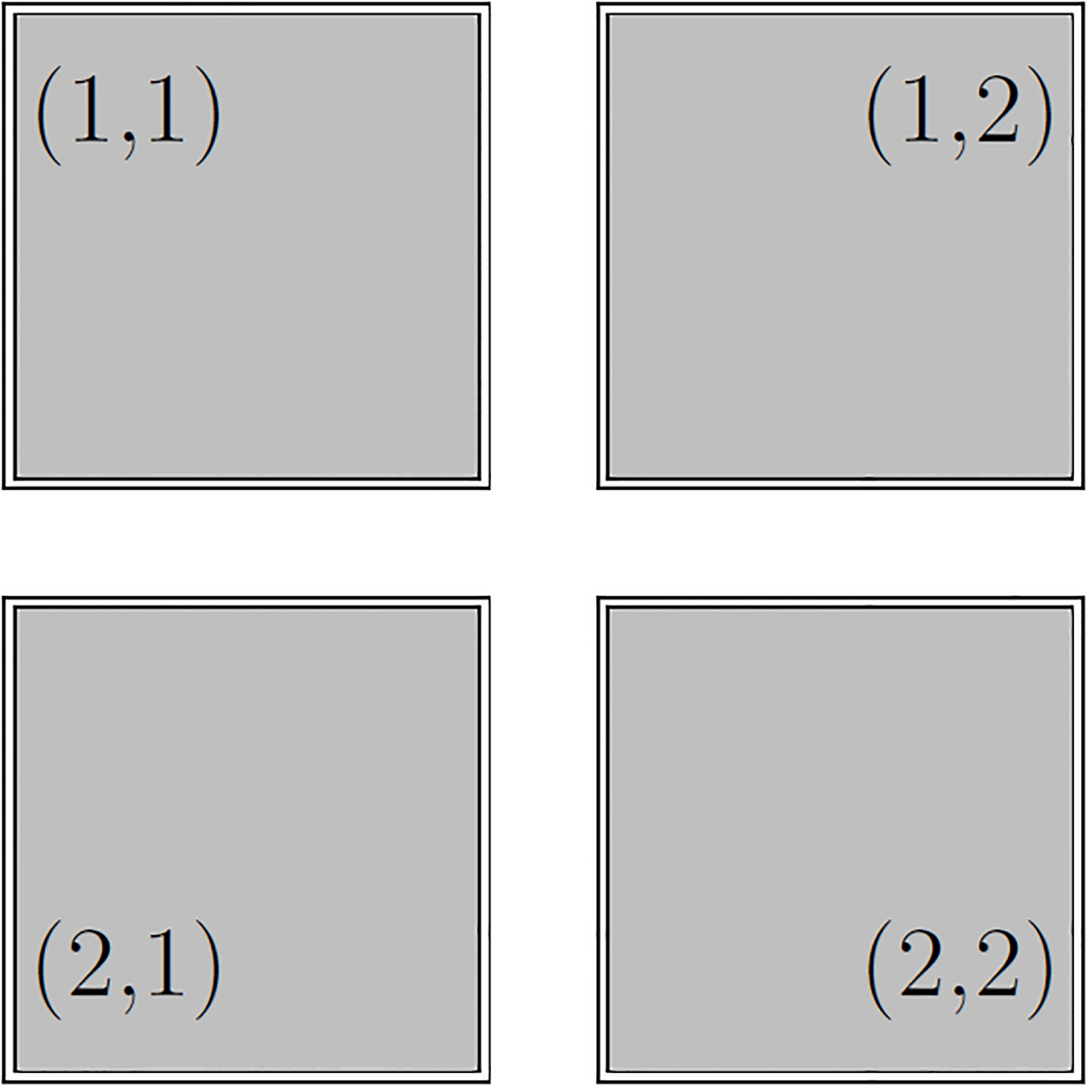
The 2 × 2 grid for multi-PTC with grid technique.

Although, multi-PTC offers an affordable resilience against brute force attack; however, it fails to ensure a high degree of resilience against such attack. Therefore, alike Knock Code [25], multi-PTC is enhanced by incorporating a grid in it. For that we consider a 2 × 2 grid, i.e., four (4) cells (*C*_*m*,*n*_), where *m* and *n* are row and column numbers, respectively; and 0 < *m*, *n* ≤ 2, as shown in Fig 3. Unlike Android-based Pattern Lock (PL) scheme [10] (which utilizes a 3 × 3 grid), we utilize smaller grid to increase the usability and to fit the grid within the limited screen of the most smart devices. There are a couple of advantages of using a large cell area, such as: *i*) it is more convenient to press on a larger area than a smaller, *ii*) larger cells can resolve fat-finger problem, *iii*) increase the memorability, and *iv*) also assist the users with weak vision in using the system.

Every cell in the grid could be considered as a limited area for providing a PTC. A user can provide *PTCs* at multiple cells with repetitions. Even it can visit the same cell in consecutive cycles. Alike multi-PTC, *τ* plays an important role here. More specifically, suppose a user provide a PTC in *C*_1,1_ and lifts his finger to provide another cycle of PTC. S/he has to start the next round within the *τ* time unit; otherwise, it would be considered as the end of the ongoing session. Again, from *C*_1,1_, a user can choose any grid to provide his next cycle of PTC; even *C*_1,1_ could be chosen. This freedom of choice resists this proposed technique from any smudge attack. The PTCs along with their cell information is jointly considered as a signature of that particular user and stored in a data structure (during registration phase) or is compared with the pre-registered signature (during authentication phase) to authenticate. Here, a pair, *φ*, is used to store the PTC of a particular cell and the identification number of that cell, i.e., *C*_*m*,*n*_ and then, it is added in a multiset, *ξ*. Later, during the authentication phase, newly given multiset, *ξ*′ is compared with the pre-registered multiset, *ξ*. The user will be given access if and only if *ξ*′ = *ξ*.

Note that, in oppose to the PTC and multi-PTC, it is not suitable for miniature devices; and hence, not screen size independent. However, it works fine on any medium to large smart devices. Again, this variant is effective in the scenarios where brute force attack is frequent and there is no restriction in retrying. Otherwise, multi-PTC provides affordable resistance against such attacks.

### Instructions for registration and authentication

**Fig 4:**
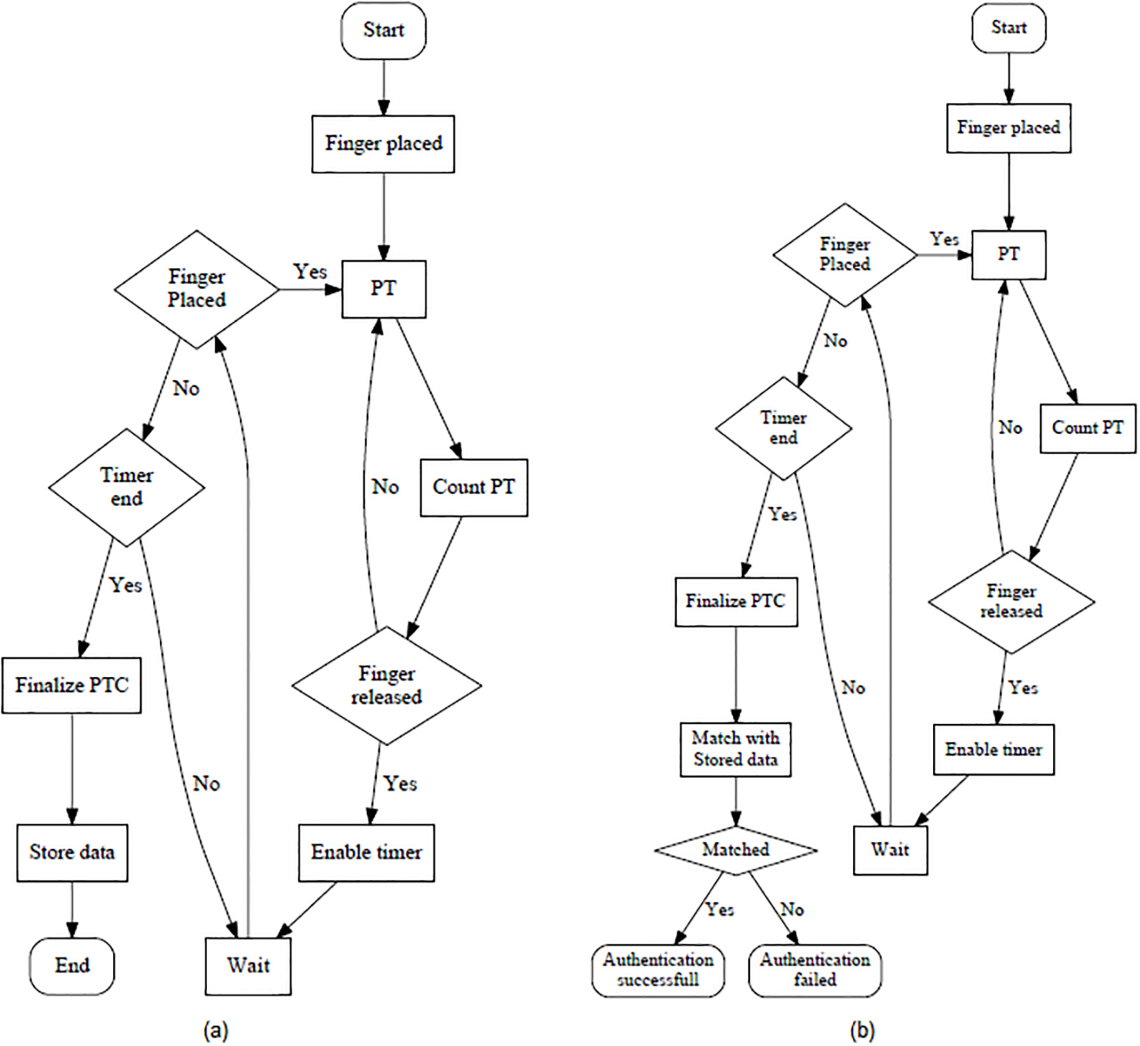
(a) Flowchart of the registration phase of the multi-PTC variant and (b) Flowchart of the authentication phase of the multi-PTC variant.

For utilizing the multi-PTC on a smart device, a user has to register a signature first. For that, the user has to launch the application. Afterwards, s/he has to place the finger within the given box on the screen as shown in Fig 1 and has to provide PTs quickly and sharply. After finishing the first cycle, the user must lift the finger from the screen. If the user desires to provide another cycle of PTC, s/he has to start the procedure within the *τ* time unit as discussed in the Proposed Scheme Section. Following this procedure, the user can repeat the PTC as many numbers of cycles as s/he wants. Any interval, *τ*′ more than *τ*, i.e., *τ*′ > *τ* would be considered as the end of the registration session. Later on, all the acquired data would be processed to extract the signature of the particular user and would be saved in *S*. The user can attempt to registrations as many times as possible until s/he is satisfied. Once the user is satisfied, s/he has to press the confirmation button; otherwise, press the repeat button. The entire registration procedure is illustrated using a flowchart in Fig 4a. In case of multi-PTC with Grid, the registration procedure is similar to that of multi-PTC except PTCs must be provided on different cells instead of random places on the screen.

Once a user is registered, the device locking system is enabled. Later on, to unlock the device, the user has to pass through an authentication session where s/he has to validate thyself by repeating the signature, which is provided during the registration session. This procedure is almost similar to the registration procedure, except the matching portion. Let us assume that the new signature provided by the user is *S*′. Now, the screen would be unlocked only when *S*′ = *S*. Both these signatures would be considered equivalent only when following two conditions are true. At first, the cardinality of both the signatures is checked. If they are equivalent, i.e., |*S*′| = |*S*|, only then the second condition is checked. In the second condition, every vector in *S*′ is compared with the corresponding vector in *S*. They are considered equivalent only if $\forall_{i}\rho_{i}^{\prime }=\rho_{i}$. The authentication process is illustrated in more details using a flowchart in Fig 4b. For multi-PTC with Grid, the procedure is same except the pre-registered *ξ* is compared with the newly provided *ξ*′, and the screen would be unlocked only when *ξ*′ = *ξ*.

## Analysis of our proposed scheme

The proposed authentication scheme has been implemented on Android Operating System and tested on a Huawei P9 Plus, which is a PST enabled device. Our application is suitable for any Android based device with similar specifications. To enable this application on other operating system requires necessary modifications. To evaluate our proposed scheme, we conduct an in-lab experiment and a comprehensive survey on 105 male and female participants of different demographics. The design of our in-lab experiment and our survey had not violated any regulation of the university’s Ethics Review Board. Prior to any experiment or any survey, a participant was asked to read and sign an informed consent form, which stated that his/her usability experience would be logged. In the form, a participant had to provide limited personal information so that later on we can trace back to him/her. Apart from that no personal information was accumulated neither during the experiment nor during the survey. Thus, all the data obtained were anonymous data. Therefore, no treatment was performed to de-identified them.

Generally, any authentication scheme has three important requirements, namely security, functionality, and usability. Therefore, in this section, we explain how the proposed scheme satisfies those requirements. In addition, we also compare our scheme with other relevant prominent schemes to demonstrate the effectiveness of the proposed scheme.

### Security analysis

In this subsection, we analyze the strength of the proposed scheme against the shoulder surfing, smudge, and brute force attacks. They are detailed below:

#### Shoulder surfing attack

To find out the resilience of the proposed scheme against shoulder surfing attacks, an in-lab experiment is performed and compares it with that of the Knock Code since it is the closest competitor of the proposed technique.

**Fig 5:**
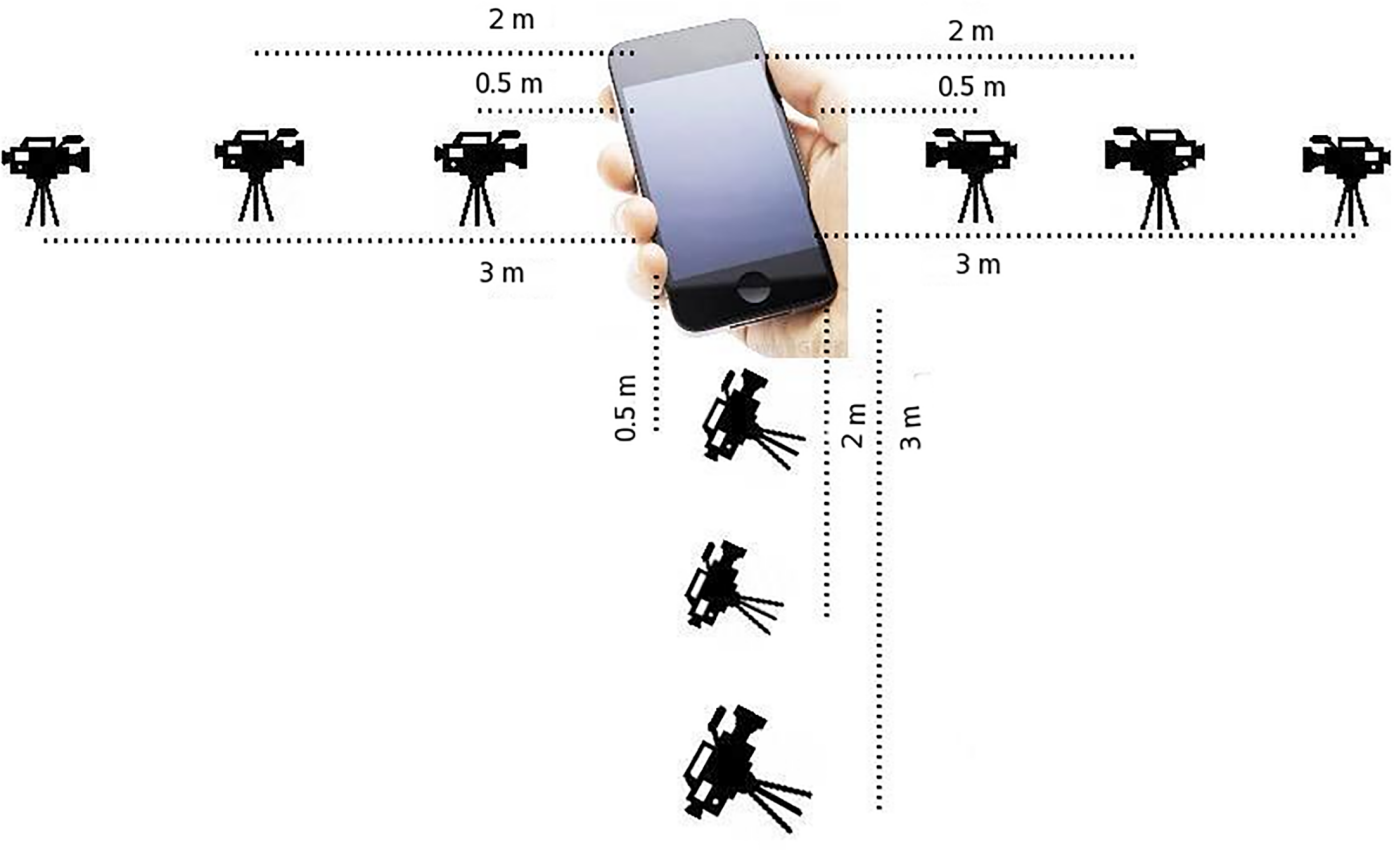
Experimental setup for discovering shoulder surfing attack on multi-PTC and Knock Code.

**Experimental setup:** In our experiment, we captured the videos of several authentication sessions using a camera of both multi-PTC and Knock Code schemes by varying several parameters as shown in Fig 5. In details, we put a camera at: *i*) three different locations: 0.5m, 2m, and 3m, *ii*) two different heights: 1.5 m (eye level height of a mature man) and 2.5m (ceiling mounted camera height), and *iii*) three different directions: left, right, and front. In all the sessions, the camera was angled towards the smartphone. The ceiling mounted camera height was taken into account only for 3m distance since from that distance, it is difficult to discover any signature from eye level height (especially, both evaluating schemes). In every session—irrespective of the scheme—a random signature was registered and it was noted down on a paper for future reference. Throughout the whole experiment, a single right-handed male model was used. During the authentication session, the phone was kept horizontal to the ground so that with little efforts an attacker can acquire the signature. After capturing the videos of all the sessions, we edited them to remove unnecessary parts. Later on, we played the recorded videos to the participants and asked them to find out the signature for all the sessions. Note that only one chance was given to the participants to discover a signature. They noted down their answers on papers and returned them to us after all the sessions were explored, which are then accumulated on an excel file.

**Results analysis**: All the results in Table 1 are computed based on the user feedbacks. As it could be observed from the table is that when the camera distance is near to the user, both the scheme has negligible or no defense against Shoulder Surfing attack. On the other hand, they attain certain levels of resilience for longer distances. Between both the schemes, multi-PTC outperforms its counterpart for all the parameters. One of the key reasons behind this is that presses are more sophisticated than knocks, and the latter could be recognized even from a long distance. Among the three directions, left side has the lowest Shoulder Surfing resilience over the other two sides for both the schemes. Since our model was right-handed, obviously he was holding the phone on the right hand, which exposed the left side and front side over right side. For that reason, at 2 m distance, right side has the most resilience against the Shoulder Surfing attack for both the schemes, i.e., 0.857 for multi-PTC and 0.67 for Knock Code. After interviewing several participants, we reveal that many participants was also observing the hand movement to determine the number of knocks and presses. The multi-PTC attains the highest resilience for the distance of 3 m when the camera was at ceiling mounted height and the direction was front, which is 0.95; whereas, for the similar parameters, Knock Code performs poorly. The reason behind this is that knocks are recognizable even from a long distance, but the presses are seldom recognizable from that distance.

**Table 1:** Experimental results of shoulder surfing attack.

Distance (m)	multi-PTC			Knock Code		
	Left	Front	Right	Left	Front	Right
0.5	0	0	0	0	0	0
2	0.37	0.5	0.875	0.37	0.5	0.67
3	0.625	0.95	0.75	0.125	0.1	0.615

#### Brute force attack

This kind of attack is possible on an authentication scheme when it offers a limited password space. In the proposed scheme, it can be found using Eq 3.

$$\begin{array}{c} P=\mu^{m}\times N^{m} \end{array}$$ (3)

where, *μ* is the highest allowable PTC in a single cycle, *m* is the number of PTC cycles, and *N* is the number of choices in cell selection. For instance, since in mono-PTC, the screen is not divided into cells, i.e., *N* = 1, and only one round of PTs is allowed, i.e., *m* = 1; *P* could be equivalent to *μ* only. If *μ* = 10, then *P* = *μ* = 10.

On the other hand, for multi-PTC, since it allows multiple cycles of PTCs and *N* = 1; *P* could be equivalent to *μ*^*m*^. Therefore, when *μ* = 10 and *m* = 5, it offers an equivalent password space to a 5-digit PIN, i.e., 10^5^, which is greater than 4-digit PIN—a common authentication scheme of many smart devices.

Again, in multi-PTC with grid, since the screen is divided into four (4) cells, a user would have four choices to provide his/her PTC in every cycle; and hence, it can offer a large password space, which is shown in Table 2 along with other two variants.

**Table 2:** The password space, P for three variants of the proposed scheme when *μ* = 10 and *N* = 4.

mono-PTC	*m*	mulit-PTC	multi-PTC with Grid
10	1	10	40
10	2	100	1600
10	3	1000	64000
10	4	10000	2560000
10	5	100000	102400000
10	6	1000000	4096000000
10	7	10000000	163840000000
10	8	100000000	6553600000000
10	9	1000000000	262144000000000

As it could be observed from the table is that multi-PTC offers an affordable resilience against the brute force attack by offering a considerably large password space for higher *m* values. However, it would fail to ensure a high degree of resilience when this kind of attack is frequent and no or limited password retrying policy is practiced. In such cases, multi-PTC with Grid would perform better due to offering a large password space even for moderate *m* values, which would take years to breach using the brute force attack.

#### Smudge attack

The Smudge attack is another prominent attack on smart devices, which occurs due to oily residues or smudges that remain on the screen or on the surface of the device as a side effect of proving a password. Accumulating and analyzing these oily smudges are easily possible through sprinkling some powder like particles over the screen or even with a camera. A recent study found that it is possible to partially unlock a screen around 92% of cases, and fully in around 68% cases [10].

Among all the variants of the proposed scheme, only multi-PTC with Grid has a possibility of experiencing such attack due to incorporating grid in the techique. However, since it permits a user to visit a cell multiple times, and thus, desponds all endeavors of an attacker to extract the information of visited cells.

### Functional analysis

In this section, we emphasize on two functionalities that are closely related to the proposed scheme and its related schemes.

**Screen size independence:** Although, at present, there are many authentication schemes in operation; however, most of them are not screen size independent as mentioned in the Introduction Section and Related work Section. This is one of the main motivations behind this work. For instance, most of the textual and graphical schemes are not screen size independent; specifically, they are not suitable for miniature smart devices. For textual schemes, they usually need a full or partial keyboard, which is not possible to fit in such devices. Again, for graphical schemes, a similar argument is appropriate since they also need to display some graphics on the screen. Among the three variants of the proposed scheme, mono-PTC and mulit-PTC are screen size independent, and could be applied on any sized smart devices.

**Short authentication time:** There exist some authentication schemes which offers higher securities, but takes a long time to authenticate; hence, are deemed not suitable for smart devices. For instance, VAP Code is a sense based technique that offers resilience against the shoulder surfing, smudge, and brute force attacks, but spends a large time in completing the authentication process. However, all three variants of the proposed scheme take considerably short times to authenticate.

### Usability analysis

For evaluating the usability of the proposed scheme, we conducted an extensive survey on 105 male and female participants of different demographics. All the participants had previous experience in using smart devices for a considerable length of time. During selecting participants, we endeavor to keep the ratio equal between male and female. Due to our concern and effort, the difference between them is now negligible; hence, it is not highlighted. We collected data through observation of the participants’ interaction with the system as well as through asking several questions to the participants. We opted for an in-person study for two reasons: *i*) since PTC requires a specific type of devices and it would be impractical to assume that all the participants have the device; and *ii*) conducting an in-person study allowed us to observe the user behavior directly.

#### Tasks

Every participant had to perform several tasks during the survey. They are discussed below:

1. Brief introduction: Since *PTC* is a new authentication scheme, it is necessary to make the participants familiar to the scheme. Therefore, before starting the survey, a brief introduction had been given to all the participants about the scheme, the registration procedure, the authentication procedure, and other related aspects.
2. Registration: At first, the system was launched to register a user. According to the given instructions in the Proposed Scheme Section, a user can repeat until desire signature was registered. When the registration was complete, the user clicked the confirmation button; otherwise, clicked the repeat button to repeat the procedure.
3. Authentication: After completing the registration, the system was enabled and it locked the screen. Then after a considerable interval, the user was asked to unlock the screen by repeating the signature that was registered before. A detail description of the authentication procedure is given in the Proposed Scheme Section. If the newly provided signature was matched with the previously registered signature, the screen was unlocked. Otherwise, a user had to repeat the procedure. Although, in many systems, three consecutive unsuccessful retries are considered as an attempt to password breach; however, for our survey, we do not apply this condition. We are so grateful to the participants that they keep patience and tried multiple times until they succeed.
4. Question & answer session: After successfully completing the registration and authentication session, the users were asked various questions to acquire their feedbacks on the proposed scheme. We are again grateful to the participants for their co-operations and answering all the questions. All the answers were noted down and they were analyzed later.

#### Results of the user study

**Fig 6:**
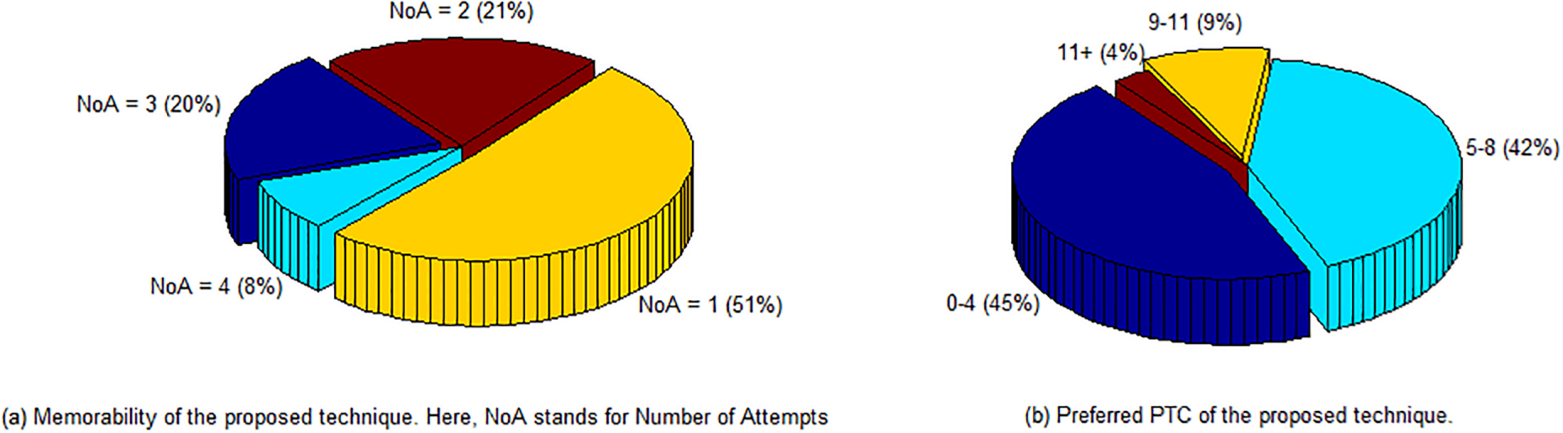
Results of the conducted survey.

This section presents the results that are acquired during the survey. To evaluate the usability of the proposed scheme, we consider two metrics, they are: *i*) memorability and *ii*) preferred PTC. Memorability is the metric which measures the quality or state of being easy to remember. Although, our proposed scheme is new, more than 70% participants were able to unlock the screen within 2 attempts; whereas, more than 90% participants were able to unlock within 3 attempts. Among the others, who needed more than 3 attempts were mostly because of insincerities during the registration phase. The survey results of memorability metric are depicted in Fig 6a, where NoA stands for Number of Attempts. All the users were able to do the registration and authentication without any difficulty, which portrays the easiness of using the system.

Fig 6b shows the percentile results of preferred PTC selection during the registration phase. As it could be seen in the figure is that around 55% participants prefer PTCs more than 4; whereas, in VC [26], only 19% participants prefer to provide VCs more than 4. It is because of convenience in PTs over sensing vibrations. The longer registration and authentication time is another factor which lowers the count for the VC. In compare to Knock Code, since during a single PTC giving cycle, a user does not have to lift the finger from the screen; it is more convenient than the counterpart where it is just opposite. Overall, the majority of the participants admitted that authentication using our proposed technique is very easy and straightforward.

### Comparison with related schemes

In this section, the proposed scheme is compared with other prominent related schemes. It is performed in terms of resistance and functionality. For this, we take five prominent related schemes into consideration along with the three variants of the proposed schemes, namely PIN [3], AlphaNumeric (AN) [4], Android Pattern Lock (APL) [10], VAP Code (VAPC) [26], and Knock Code (KC) [25].

Table 3 lists the resistance and functionality comparison of prominent related schemes along with the three variants of the proposed scheme. As could be observed from the table is that PIN, AN, and APL schemes are vulnerable to shoulder surfing, brute force, and smudge attacks in the range of medium to high. On the other hand, VAPC and KC have resistance from low to medium for those attacks. Again, all the three variants of the proposed scheme offer low to lower medium resistance for the shoulder surfing and smudge attacks. However, in case of the brute force attack, mono-PTC has high vulnerability, multi-PTC has medium vulnerability, and multi-PTC with Grid has low vulnerability. Again, from the table, it could be observed that most of the schemes are not screen size independent. Only KC, mono-PTC, and multi-PTC could be applied also in miniature devices. From the above discussions, we can come to a conclusion that VAPC and KC are the closest competitor of the proposed scheme.

**Table 3:** Comparison of prominent related schemes with the three variants of the proposed scheme.

Attack/Function	PIN	AN	APL	VAPC	KC	mono-PTC	multi-PTC	multi-PTC with Grid
Shoulder surfing	H	H	H	L	M	LM	LM	LM
Brute force	H	M	M	L	L	H	M	L
Smudge	M	M	H	L	L	L	L	L
Screen Size Independence	N	N	N	N	Y	Y	Y	N
Short Authentication Time	Y	Y	Y	N	Y	Y	Y	Y

L—Low, LM—Lower Medium, M—Medium, H—High, N—No, Y—Yes.

Although VAPC offers the high degree of resilience against three prominent attacks that are taken into account in this comparison; however, it is not screen size independent and takes a long time during authentication phase as demonstrated in the Related work Section with an example. On the other hand, all the variants of the proposed scheme are more resilient against shoulder surfing attacks than KC, which is demonstrated in the Shoulder surfing attack Subsection by performing an experiment. Consequently, if we consider the trade-off among all the related schemes in terms of resistance and functionality, our proposed scheme has better efficiency than others.

## Validity threats

Several validity threats can be associated with our in-lab experiment and our survey that we have conducted. Among them, significant threats are identified and mentioned below along with the steps that we have taken into account to mitigate them on the acquired results.

- Firstly, the choice of the device poses an essential threat since there are several other devices available in the market with the similar specifications. Note that, our proposed scheme has been implemented and tested on the Huawei P9 Plus device as mentioned earlier. However, in favor of our selection of the device, we would like to argue that the press intensity value has been acquired by calling an Android function, which is device independent. Therefore, our proposed scheme is applicable on any PST enabled Android device. On the other hand, it requires a considerable amount of modifications in terms of implementation to adopt to other PST enabled devices with different operating system.
- Secondly, data acquisition from a fixed box or area on the screen is another important threat. We utilize this box to expel any confusion that may arise in deciding where to press. Again, we have opted this since from our investigation, we discover that—irrespective of the area—a PT provides similar haptic feedback and elicit a similar set of responses depending on the intensity of the press. Hence, we would like to argue that the press intensity values would not differ due to changing or expanding the data acquisition area.
- Thirdly, the parameters those have been chosen to conduct the experiment are indispensable threats, because any parameter tuning may produce a different set of results. However, in our experimental setup, the parameters were selected in such a way that they may bring off the vulnerabilities of both the tested schemes (i.e., Knock Code and multi-PTC) without favoring any one of them. For instance, during all the experiments, the device was kept horizontal to the ground since it is the most vulnerable position for the Shoulder Surfing attack. Any change in the position would result in higher defense against the attack for both the schemes.
  Again, instead of field-based experiments, we conducted lab-based experiments where we recorded videos of various authentication sessions. Later on, we played them all on a standard laptop monitor to the participants and acquired their feedbacks. Again, to bring off the vulnerabilities of both the schemes, such setup is adopted. During a filed-based experiment, several environmental factors (such as movement, conversation, light, and so on) would influence the results; whereas, in lab-based experiments, those were absent. Therefore, the participants were able to recognize the signatures oftenly for various parameters.
- Fourthly, in our survey, we selected only those participants who have a considerable duration of experiences in using smart devices, which also could be considered as a threat. However, we would like to lay following arguments in favor of our selection: *i*) experience users would require minimum briefing to introduce the proposed scheme, *ii*) experience participants would spend lower time in registration and authentication than others, and *iii*) they could give us more productive suggestions and feedbacks, which would later assist us in improving the scheme.
- Finally, the choices of performance metrics for evaluating the effectiveness of the proposed scheme can also pose as threats. In our case, although we consider two metrics, namely *i*) memorability and *ii*) preferred PTC; but other metrics also exist. Among the selected metrics, memorability is a well-known and well-established metric that tells the easiness in remembering a scheme. Any complex scheme may increase the security of the system, but people would only embrace this scheme if it is easy to remember. Hence, the choice of this metric is appropriate. Again, the second metric is related to the proposed scheme, which is necessary to understand user behavior in providing the PTC.

## Conclusion

In this paper, we present a new password-based authentication scheme, which transforms the PT of the PST enabled smart devices to a code, named PTC. We introduce three variants of the proposed scheme, namely mono-PTC, multi-PTC, and multi-PTC with Grid. Among them, former two variants are screen size independent and also offers resilience against the Shoulder Surfing and smudge attacks, since mono-PTC offers a limited password space; hence, it is more vulnerable than the other. Conversely, multi-PTC offers an affordable defence against such attack by allowing a user to repeat PTC for multiple cycles. Again, although multi-PTC with Grid is not screen size independent, but it has resilience against all three prominent attacks mentioned earlier. The effectiveness of the proposed scheme is evaluated using an in-lab experiment and a comprehensive survey on 105 male and female participants. Our experiment shows that the proposed scheme offers a higher resilience against Shoulder Surfing attack over the Knock Code. The responses from the participants are also analyzed and found positive; and they admit that the proposed scheme is easy to use. We also compare our proposed scheme with five prominent related schemes in terms of resistance and functionality. From the comparison, we can conclude that our proposed scheme is more efficient than others since it shows better trade-off.

## Supporting information

S1 File Sample press data set. (DOCX)
