# Supplementary material for: Press touch code: A finger press based screen size independent authentication scheme for smart devices
Source: PLoS One. 2017 Oct 30;12(10):e0186940. doi: 10.1371/journal.pone.0186940 (PMC5662178; doi:10.1371/journal.pone.0186940)
Supplement: S1 File — (DOCX) [file pone.0186940.s001.docx]

0.000686656,0.004180972,0.010986496,0.048447393,0.068284124,0.10215915,0.1132372,0.14366369,0.16229495,0.17642482,0.20466927,0.21576257,0.2409247,0.25516137,0.26413366,0.28926528,0.30037385,0.33411154,0.34309912,0.38202488,0.4138857,0.45491722,0.46218053,0.49338523,0.50952923,0.51841,0.5308461,0.5342794,0.537087,0.5371023,0.53290606,0.50238806,0.48830396,0.4350805,0.40277714,0.35988402,0.3303273,0.32289615,0.31218433,0.30948347,0.30785078,0.30678263,0.30714884,0.31313038,0.34335852,0.3803769,0.38551918,0.39632258,0.41345847,0.41744107,0.42253757,0.42325476,0.42279696,0.42078277,0.382681,0.37524986,0.34354162,0.32343024,0.31259632,0.2964828,0.2806592,0.27728695,0.27493706,0.272343,0.2719005,0.27278554,0.27547112,0.3216907,0.33568323,0.38307774,0.41570154,0.42307165,0.45188066,0.46305028,0.47258717,0.47489128,0.47632563,0.47609675,0.4704509,0.43920043,0.42827496,0.38069734,0.34857708,0.3083238,0.30220494,0.27397573,0.2628977,0.25392538,0.23988709,0.22949569,0.22771038,0.22626078,0.2262913,0.22691691,0.24022278,0.27476922,0.31903562,0.3528954,0.3990997,0.40531015,0.41976044,0.43608758,0.441413,0.4470741,0.447776,0.4470741,0.4447242,0.40006104,0.38892195,0.3561303,0.31631953,0.28894484,0.2701457,0.26665142,0.26399633,0.26141757,0.2610361,0.26182956,0.2640879,0.30432594,0.3152819,0.36266118,0.3937438,0.39987794,0.42552835,0.43163195,0.43814754,0.4394293,0.43961242,0.4383917,0.43439382,0.39108872,0.3573663,0.31496146,0.307805,0.28218508,0.27122912,0.2589456,0.25241473,0.24919508,0.24858473,0.24856947,0.24936293,0.25639734,0.27597466,0.29018083,0.320943,0.3510033,0.36229494,0.36951247,0.37798125,0.38059053,0.38274205,0.38287938,0.38252842,0.37781337,0.36668956,0.32814527,0.29739833,0.27809566,0.26717022,0.25635156,0.24290837,0.23776607,0.2322118,0.23108263,0.23093003,0.23218128,0.26796368,0.27521172,0.30844587,0.35091174,0.35696957,0.38191807,0.38913557,0.39816892,0.4004425,0.40196842,0.40195316,0.40109864,0.38764018,0.3562066,0.3300679,0.32118714,0.29980928,0.2872816,0.28033876,0.27855346,0.27747005,0.27785152,0.28564888,0.3185626,0.32971695,0.37813383,0.41022354,0.43758297,0.446479,0.46253148,0.46588847,0.46915388,0.46930647,0.46878767,0.45593956,0.4206302,0.38181123,0.3481956,0.30565345,0.29951933,0.2772717,0.26840618,0.26228732,0.2554513,0.25363547,0.25258258,0.2529946,0.26091403,0.29423973,0.3084001,0.35211718,0.3838102,0.40003052,0.4087129,0.41661707,0.41849393,0.41939422,0.4187991,0.41722745,0.3795529,0.37068743,0.3312276,0.3004349,0.28722057,0.27627984,0.26752117,0.25691617,0.2538796,0.25148395,0.25143817,0.25520715,0.28644237,0.3004349,0.35387197,0.3849546,0.41211566,0.41937897,0.42917526,0.43126574,0.43266958,0.43244067,0.43118945,0.3966125,0.38794538,0.34619668,0.3143206,0.27554742,0.27032882,0.2399939,0.22000457,0.21110857,0.1858091,0.17689784,0.1537499,0.14502174,0.12904555

0.013839933,0.04742504,0.07775997,0.14024568,0.17544824,0.25397116,0.29680324,0.3411002,0.42716107,0.46985582,0.5490959,0.5838712,0.64869153,0.6559701,0.69047076,0.71458,0.72062254,0.73127335,0.72156864,0.6882887,0.6407263,0.62121004,0.5784848,0.5451591,0.5044022,0.49835965,0.47624934,0.46555275,0.534432,0.5644617,0.627039,0.658259,0.6727245,0.72732127,0.758114,0.77520406,0.7945983,0.8107729,0.8257572,0.83289844,0.8430457,0.84362555,0.843534,0.8425727,0.8399634,0.7996338,0.74714273,0.715404,0.6751202,0.66982526,0.6479591,0.63448536,0.6271153,0.638056,0.6818189,0.7134051,0.75045395,0.7698024,0.78593117,0.8000305,0.81345844,0.8201114,0.8316167,0.82653546,0.81554896,0.79264516,0.7782406,0.76932937,0.7516899,0.7456321,0.73798734,0.7481346,0.76252383,0.7822232,0.79961854,0.8108034,0.8324254,0.851133,0.8624247,0.8718242,0.86996263,0.85818267,0.84942394,0.8332189,0.8183108,0.8068971,0.7985046,0.8201419,0.84008545,0.85348284,0.86228734,0.8805676,0.890959,0.90090793,0.91090256,0.9084459,0.8965744,0.88912797,0.8787518,0.8818189,0.8938125,0.91146713,0.9312886,0.94026095,0.95140004,0.94929427,0.9257496,0.91847104,0.90957505,0.8997787,0.88912797,0.8909285,0.900267,0.8986801,0.888716,0.8778363,0.859617,0.84623486,0.8263066,0.8175326,0.80485237,0.79880977,0.78832686,0.7789883,0.78028536,0.8079652,0.8190738,0.8300755,0.850309,0.8707866,0.88552684,0.89393455,0.9011368,0.8759289,0.86172277,0.82236975,0.80807203,0.77752346,0.74126804,0.72492564,0.7104448,0.70160985,0.6845655,0.67852294,0.6694743,0.67037463,0.68195623,0.71400017,0.75306326,0.76021975,0.7903563,0.80972,0.8331731,0.8442054,0.8550393,0.8624094,0.8758831,0.88108647,0.8909743,0.88316166,0.85111773,0.8081941,0.7886015,0.7459678,0.71042955,0.67115283,0.63607234,0.6217136,0.58930343,0.549218,0.51165026,0.50707257,0.48183414,0.4728313,0.46189058,0.44585335,0.43962768,0.42906845,0.41916534,0.4548867,0.4692912,0.5414664,0.5748989,0.79375905,0.85848784,0.87254137,0.91639584,0.94837874,0.9556268,0.9600519,0.971664,0.9801175

0.05120928,0.083299,0.11635004,0.15176623,0.19020371,0.23152514,0.32162967,0.3699092,0.46764323,0.5160296,0.6080873,0.6506905,0.6897841,0.7568017,0.78696877,0.82555884,0.8306859,0.84403753,0.850248,0.8348211,0.7977874,0.72674143,0.69117266,0.6200351,0.5854429,0.55329216,0.5139086,0.5030442,0.4889601,0.52622265,0.5583734,0.61957735,0.6499275,0.7021134,0.7215839,0.7612726,0.7925841,0.820737,0.8314946,0.81466395,0.78416115,0.7394369,0.7197223,0.68900585,0.65093464,0.63558406,0.6246891,0.61847866,0.6097963,0.6329137,0.6646677,0.71271837,0.7321126,0.7635767,0.8044709,0.84045166,0.8514992,0.8604562,0.8722362,0.8802014,0.8798047,0.8333257,0.8012207,0.7366293,0.70379186,0.6388495,0.6081178,0.59362173,0.55138475,0.5318837,0.5258412,0.51781493,0.55101854,0.5704433,0.6230564,0.65603113,0.7033036,0.71219957,0.75259024,0.7667353,0.77557033,0.7829251,0.79121083,0.78988326,0.75872433,0.704509,0.670985,0.6382238,0.573724,0.5424277,0.4900435,0.47904173,0.44847792,0.43840694,0.4298314,0.47933164,0.5111925,0.56156254,0.59531546,0.62533,0.66945904,0.67833984,0.7129168,0.7262074,0.7320058,0.6955825,0.67617303,0.61316854,0.59867245,0.5326314,0.5214465,0.48767835,0.44669262,0.42775616,0.45479515,0.48566416,0.5481651,0.579736,0.6305333,0.6640879,0.7139086,0.7394522,0.7528954,0.7603876,0.7222858,0.7078813,0.67373157,0.6214542,0.61025405,0.56783396,0.5369802,0.52449834,0.517113,0.5072557,0.5433127,0.5627985,0.61519796,0.6480354,0.69556725,0.7066148,0.7383383,0.7639277,0.77126724,0.783856,0.7904326,0.7886778,0.7744564,0.72684824,0.6924849,0.6512093,0.6166934,0.5667506,0.5407797,0.51773864,0.5054093,0.49983978,0.50045013,0.54433507,0.5762875,0.6380102,0.6525063,0.71940184,0.73038834,0.77062637,0.7905394,0.8155032,0.82204926,0.81997406,0.80035096,0.748394,0.7151751,0.66459143,0.6305333,0.5886778,0.55565727,0.5324788,0.52161443,0.51630425,0.5233997,0.5950408,0.6276799,0.68958575,0.70402074,0.73794156,0.78220797,0.80268556,0.81057453,0.81083393,0.8089265,0.80164796,0.7674067,0.72260624,0.687068,0.6418555,0.6080873,0.56241703,0.55526054,0.54076445,0.51947814,0.5143359,0.5097581,0.5115282,0.52596325,0.5962921,0.6284123,0.6606088,0.72234684,0.74192417,0.78825057,0.8196689,0.8453193,0.85265887,0.86150914,0.87092394,0.87295336,0.87415886,0.8737774,0.8631571,0.82816815,0.7644465,0.73270774,0.70029753,0.6336614,0.59984744,0.5318532,0.4982681,0.4329595,0.4007477,0.3686122

0.03590448,0.046906233,0.078080416,0.08526742,0.11755551,0.1286946,0.16363776,0.22534524,0.25763333,0.32867932,0.3682765,0.45423058,0.4991989,0.54517436,0.63506526,0.6772106,0.7544518,0.78892195,0.82861066,0.8601663,0.87653923,0.8838178,0.893843,0.89820707,0.89781034,0.8929427,0.88844126,0.88316166,0.8699016,0.86462194,0.8555886,0.85288775,0.8510872,0.84969866,0.85081255,0.8569161,0.8891585,0.93243307,0.9434501,0.9614252,0.96438545,0.9688258,0.9706569,0.97372395,0.9804227,0.9820706,0.98135346,0.9773861,0.9254597,0.893843,0.87959105,0.83576715,0.81625086,0.8016327,0.7943389,0.78641945,0.78452736,0.7831845,0.78327614,0.78423744,0.8256199,0.84521246,0.89724576,0.9283742,0.96641487,0.9685054,0.97198445,0.97354084,0.97492945,0.9773098,0.97837794,0.98028535,0.98113984,0.9827268,0.983444,0.9843595,0.9835508,0.97630274,0.9087663,0.894316,0.839765,0.80817884,0.7688411,0.76586556,0.7603571,0.7603876,0.7659571,0.79822993,0.8500191,0.8612039,0.90835434,0.94140536,0.9502098,0.9603723,0.9634089,0.9681392,0.97000074,0.97323567,0.97463953,0.9763485,0.9756619,0.9710689,0.9289235,0.89575034,0.8493934,0.84045166,0.80381477,0.79649043,0.78933394,0.7888609,0.7900511,0.79446095,0.848188,0.88250554,0.9289998,0.93785,0.96891737,0.97235066,0.97640955,0.97752345,0.9795529,0.98046845,0.97961396,0.9750668,0.94007784,0.87353325,0.84119934,0.77785915,0.74683756,0.69501793,0.686244,0.6611734,0.65603113,0.65215534,0.6529946,0.6998245,0.73972684,0.8214084,0.8617533,0.93742275,0.96870375,0.9705348,0.9736324,0.97500575,0.97737086,0.978439,0.9803464,0.9812009,0.98275733,0.9834745,0.98416114,0.981033,0.950103,0.9006333,0.88630503,0.8486915,0.83447015,0.8226444,0.8195926,0.81829554,0.8180972,0.81971467,0.87776,0.9213245,0.96368355,0.96627754,0.9683833,0.97187763,0.97343403,0.97607386,0.979263,0.9801938,0.98187226,0.9826505,0.9833677,0.98467994,0.9844053,0.94357216,0.90562296,0.8370031,0.8170138,0.78522927,0.7727016,0.7681239,0.76542306,0.7663386,0.8157473,0.85391015,0.937026,0.9685054,0.97198445,0.97354084,0.97492945,0.9773098,0.97837794,0.98028535,0.98113984,0.9827268,0.983444,0.98475623,0.9853513,0.98593116,0.98619056,0.98356605,0.93838406,0.9060502,0.8776074,0.8644846,0.8634165,0.8636759,0.9027695,0.93495077,0.9694972,0.97128254,0.9742885,0.9755703,0.976791,0.9789426,0.9798886,0.9807889,0.9824063,0.9838407,0.9845121,0.98571754,0.9862821,0.98680097,0.9877928,0.98825055,0.9833677,0.9465171,0.87087816,0.8337377,0.7800412,0.74407566,0.71308464,0.66671246,0.62894636,0.6125887,0.5895323,0.58434427,0.56827646,0.5609369,0.554757,0.5495537,0.5419089,0.5388571,0.538674,0.55075914,0.59342337,0.64310676,0.7689174,0.8283055,0.9406577,0.96043336,0.9660792,0.96818495,0.9717098,0.97328144,0.97593653,0.9771267,0.9782101,0.98013276,0.98098725,0.98139924,0.97689784,0.89825284,0.8615244,0.8272221,0.7611353,0.75004196,0.70637065,0.69765776,0.69338524,0.6934768,0.73871976,0.784741,0.84255743,0.9507591,0.9569085,0.9638209,0.9663996,0.97033644,0.97198445,0.97492945,0.9761807,0.9773098,0.97935456,0.98028535,0.98194855,0.9827268,0.9841306,0.9843595,0.9489433,0.9134661,0.87821776,0.8121462,0.8034333,0.76974136,0.7676509,0.76778823,0.7738613,0.8707866,0.9181048,0.9590448,0.9651179,0.96743727,0.971252,0.97283894,0.974258,0.97891206,0.9798581,0.9807736,0.98239106,0.98312354,0.98449683,0.9851072,0.98475623,0.952636,0.8762188,0.8378118,0.80177003,0.76351565,0.7524224,0.74517435,0.7453117,0.7904326,0.8326085,0.8768139,0.9616693,0.96461433,0.9690242,0.97084004,0.97390705,0.9752804,0.97651637,0.9796445,0.98139924,0.98220795,0.98364234,0.9843137,0.9855344,0.986099,0.9845121,0.94409096,0.89910734,0.8153048,0.77840847,0.7414359,0.73611045,0.73369956,0.73466086,0.8104219,0.8522011,0.89784086,0.9553674,0.959823,0.96578926,0.9680629,0.9716945,0.9732662,0.97593653,0.98013276,0.98098725,0.9825895,0.98330665,0.9846189,0.98522925,0.9863737,0.9868925,0.98739606,0.9667811,0.92774856,0.9008164,0.8809339,0.87653923,0.8762951,0.8970779,0.9372549,0.96862745,0.97209126,0.9735561,0.97619593,0.9773251,0.9783932,0.9803006,0.9827421,0.98345923,0.9847715,0.9853666,0.9864958,0.9870146,0.9879759,0.98843366,0.9885557,0.9424582,0.9024033,0.8517891,0.8204776,0.81190205,0.8106661,0.81104755,0.8140993,0.8461738,0.91371024,0.9507286,0.9607843,0.96379036,0.9684596,0.9703059,0.9735103,0.97615016,0.9783474,0.97932404,0.9811093,0.98191804,0.98341346,0.9841001,0.984329,0.9443046,0.9028,0.8270085,0.7928588,0.7508049,0.73101395,0.719646,0.7170062,0.71607536,0.71615165,0.7181659,0.7833219,0.8210727,0.9068742,0.9518883,0.9617151,0.9646601,0.96701,0.9708858,0.97529566,0.97651637,0.9786832,0.9796445,0.98139924,0.98220795,0.98294044,0.9840238,0.95347524,0.8731518,0.83051807,0.7595788,0.74509805,0.7053941,0.6964065,0.69195086,0.6866255,0.68593884,0.6887617,0.7268635,0.8143282,0.86439306,0.9583886,0.96185243,0.9647822,0.9691615,0.9709621,0.9725643,0.9753567,0.9776913,0.9787442,0.9797055,0.9814603,0.98225373,0.9836881,0.9843595,0.9844663,0.9703059,0.8899672,0.8564431,0.8226597,0.8180819,0.8153811,0.8140383,0.8152285,0.8766155,0.91943234,0.9630732,0.9656977,0.9679713,0.97172505,0.97328144,0.97593653,0.9771267,0.9791867,0.98013276,0.9818112,0.9825895,0.98330665,0.9846189,0.98522925,0.9863737,0.9868925,0.98736554,0.9859159,0.97541773,0.9715572,0.97029066,0.9707332,0.97235066,0.9751736,0.97640955,0.9785916,0.9795529,0.9813077,0.9821164,0.9828489,0.9842222,0.9848478,0.9854429,0.9865568,0.98754865,0.9880217,0.9884642

0.010040436,0.029755093,0.049698636,0.080704965,0.09170672,0.12487984,0.13891813,0.17439537,0.18519875,0.19244678,0.20103762,0.2043183,0.19237049,0.18336767,0.16136415,0.15265125,0.14535744,0.13556115,0.12971695,0.1301442,0.14590676,0.15114062,0.16054016,0.15748836,0.14734112,0.1326009,0.12518501,0.1179675,0.10751507,0.135729,0.14978255,0.1586633,0.17347983,0.18103303,0.17674525,0.16169986,0.15278858,0.13659877,0.12938124,0.11801328,0.10844587,0.11300831,0.14683756,0.16630808,0.17351034,0.18092622,0.1914702,0.18692301,0.1671168,0.15835813,0.14959945,0.13714808,0.128954,0.1301442,0.14827192,0.16263066,0.18268101,0.19505608,0.2005188,0.1991455,0.17175555,0.16275273,0.15158312,0.13182269,0.124559395,0.11926451,0.12213321,0.15033188,0.1613489,0.1702449,0.1836881,0.191104,0.18602273,0.17984283,0.16371405,0.15642023,0.13960479,0.18326086,0.1994049,0.21547265,0.22001983,0.21574731,0.19571221,0.18471046,0.16720836,0.15655756,0.14970626,0.15008774,0.16166934,0.17071794,0.16849013,0.15497063,0.14766155,0.1369192,0.12959488,0.13057145,0.1584802,0.16583505,0.17676051,0.19073777,0.20021363,0.19879454,0.20097658,0.2350042,0.24119936,0.2506752

0.00202945,0.0031433585,0.0045471885,0.0071717403,0.012359807,0.026352331,0.040817883,0.07635614,0.11169604,0.13144122,0.14262608,0.17735562,0.18818952,0.20613413,0.21509117,0.22583352,0.22932784,0.23198292,0.23541619,0.23613337,0.23643854,0.23604181,0.23378347,0.23170824,0.22618449,0.22383459,0.22218663,0.22049287,0.22026399,0.22099641,0.22224766,0.22877851,0.2339971,0.24377814,0.24721141,0.24985123,0.25255206,0.25316244,0.2533303,0.24892043,0.23717098,0.2299382,0.22995345,0.23178454,0.24711986,0.25824368,0.27243456,0.28580147,0.29095903,0.29723048,0.29881743,0.29098955,0.2727703,0.26186007,0.25305563,0.24327458,0.24020752,0.23689632,0.23625544,0.23630121,0.24200809,0.24818799,0.2569772,0.27286184,0.27740902,0.28467232,0.28651866,0.28755626,0.28722057,0.28332952,0.27818722,0.267216,0.25830472,0.24641794,0.24129091,0.24019226,0.23956664,0.23990235,0.241001,0.24306096,0.2546273,0.26176852,0.27362478,0.28093386,0.28273442,0.28484017,0.28484017,0.28436714,0.2810254,0.27702755,0.25517663,0.24399176,0.22894636,0.22446021,0.2187686,0.217319,0.21646449,0.21602197,0.21625085,0.21991302,0.22462806,0.22700846,0.23103686,0.23208973,0.23251697,0.2322118,0.23123522,0.22572671,0.21966888,0.2035706,0.19732967,0.18820478,0.18558022,0.18413062,0.18298618,0.18306248,0.18512246,0.20944533,0.2203708,0.24284734,0.24887465,0.2540322,0.25513086,0.2554818,0.25497827,0.25102618,0.24646372,0.24022278,0.22763409,0.22043183,0.21979095,0.21994354,0.22099641,0.23494317,0.24585336,0.26051727,0.26449987,0.26968795,0.2706035,0.27064928,0.26973373,0.25441366,0.24541084,0.23352407,0.23088425,0.22829023,0.22790875,0.22819868,0.23012131,0.23344778,0.23503472,0.23752193,0.23808652,0.23788816,0.23682001,0.2299382,0.22259861,0.21382467,0.20856032,0.2009308,0.20117494,0.2104677,0.22172885,0.23166247,0.2332189

0.0022735943,0.004821851,0.0071869995,0.011108568,0.018356603,0.03257801,0.047028307,0.09312581,0.13196002,0.17450218,0.2865797,0.35555047,0.5137255,0.6023041,0.7690547,0.8442359,0.95553523,0.9633021,0.9681697,0.97003126,0.9732662,0.97467005,0.98013276,0.9786679,0.9408713,0.8950942,0.86158544,0.8506447,0.8164187,0.80672926,0.80375373,0.8537728,0.9010605,0.9559625,0.96035707,0.97164875,0.980972,0.98179597,0.9832914,0.98397803,0.98463416,0.9760281,0.938735,0.8865339,0.86652935,0.8563668,0.88770884,0.9262684,0.96575874,0.96803236,0.9716793,0.97917145,0.9801175,0.90952927,0.87733275,0.83167773,0.8281834,0.8307622,0.9191577,0.9595789,0.96557564,0.9716335,0.97320515,0.9758755,0.97708094,0.9781643,0.98010224,0.98255897,0.9832761,0.9846189,0.98522925,0.9858091,0.9863737,0.9552148,0.9407187,0.89260703,0.8731823,0.86793315,0.9221637,0.96107423,0.9703975,0.97726405,0.97833216,0.98023957,0.98109406,0.98268104,0.9833982,0.98471045,0.9853208,0.98590064,0.98645,0.9874571,0.9879301

0.03418021,0.0539559,0.085435264,0.1394522,0.17355612,0.207599,0.28291753,0.32310978,0.40131226,0.43801022,0.47896543,0.50966656,0.5127184,0.49884793,0.46729228,0.43894103,0.42548257,0.4197757,0.42310217,0.45525292,0.5214465,0.5413291,0.5829709,0.6130007,0.620325,0.61858547,0.6076143,0.5413291,0.5270619,0.48975357,0.4789044,0.48003358,0.53235674,0.5693904,0.6377966,0.668101,0.7108568,0.7211414,0.71635,0.69910735,0.7080491,0.7802243,0.8107424,0.8269627,0.83137256

0.062149998,0.0964065,0.1284352,0.14814985,0.17900358,0.1841764,0.19145495,0.20073244,0.19990845,0.1723659,0.16142519,0.14532693,0.13908598,0.12784009,0.119111925,0.1520409,0.18472572,0.25090408,0.2653544,0.29581138,0.30156404,0.30373085,0.29442284,0.26413366,0.23944457,0.22850385,0.20901808,0.18590066,0.1750515,0.15878539,0.1493553,0.18348974,0.21881437,0.2530709,0.28349736,0.29478905,0.29810026

0.05368124,0.06085298,0.09303426,0.103852905,0.13649195,0.14735638,0.18553445,0.20515755,0.21243611,0.22500953,0.23021287,0.22977035,0.19227894,0.18124667,0.16112001,0.15370412,0.14653239,0.13850614,0.13322651,0.13348593,0.13582055,0.18184176,0.21383993,0.27907225,0.31445792,0.3891203,0.4271916,0.46456093,0.5334402,0.5534142,0.5907683,0.6016327,0.61074233,0.5969787,0.52567333,0.4909285,0.4220035,0.38835737,0.3254139,0.31128404,0.27867553,0.23825437,0.21321431,0.20453192,0.19922179,0.18931869,0.19070725,0.23245594,0.30104524,0.3374838,0.4147097,0.4543374,0.4936904,0.5678492,0.6013733,0.6430915,0.67435724,0.6840009,0.6232548,0.5884184,0.5147326,0.47826353,0.40773633,0.37430382,0.32948807,0.29698634,0.2882887,0.25732815,0.24106203,0.22140841,0.2152285,0.2066682,0.22568093,0.25975433,0.32706186,0.3633936,0.43944457,0.47858396,0.5172503,0.5899138,0.6231022,0.6650492,0.6971389,0.7123674,0.72352177,0.69416344,0.66131073,0.58956283,0.5515831,0.47701228,0.44072634,0.40593576,0.34117648,0.33000687,0.2881056,0.25792325,0.24771497,0.23527886,0.22919051,0.26991683,0.3063554,0.38429847,0.42662698,0.46958113,0.5567102,0.59882504,0.6769512,0.7125963,0.7650721,0.79674983,0.8358587,0.84225225,0.84388494

0.003418021,0.00999466,0.0568246,0.07132067,0.10840009,0.18381017,0.22661173,0.3243305,0.37785915,0.48622873,0.5381094,0.5861906,0.66849774,0.70168614,0.7178912,0.7239948,0.6915236,0.65816736,0.5868162,0.5506981,0.4783093,0.44341192,0.3782254,0.3587854,0.31883726,0.28853285,0.26956588,0.25893036,0.26015106,0.29109636,0.36430916,0.4063325,0.450309,0.5386435,0.5812009,0.65809107,0.6920882,0.7421378,0.75458914,0.76240176,0.75988406,0.69169146,0.657496,0.58724344,0.550988,0.5161822,0.45223165,0.43241015,0.38486305,0.35249865,0.32379645,0.31276417,0.28986037,0.27980468,0.2764935,0.31169605,0.38280308,0.4269627,0.51338977,0.5520409,0.5912718,0.66324866,0.6942855,0.7375601,0.7689021,0.7743801,0.77593654,0.77674526,0.77679104,0.7753109,0.731487,0.6971084,0.628336,0.59464407,0.5259022,0.49311054,0.46118867,0.4160067,0.38146028,0.33186847,0.3256428,0.2898451,0.26381323,0.25868618,0.30228123,0.3458915,0.43569085,0.48197147,0.5285725,0.6187686,0.66053253,0.7337301,0.7715877,0.7858854,0.7930724,0.7997864,0.8006409,0.80065614,0.7924163,0.7556878,0.6855726,0.6505684,0.5807279,0.54735637,0.51541924,0.46399635,0.45513085,0.4197757,0.40834668,0.43920043,0.47621882,0.55950254,0.6031891,0.69379723,0.7326162,0.796582,0.8360418,0.8473335,0.8545968,0.8585031,0.8599832,0.8581369

0.022034027,0.055451285,0.11795224,0.15223926,0.18980697,0.27502862,0.32071412,0.4120699,0.45448998,0.5271382,0.5414817,0.5719539,0.5802854,0.5778744,0.5248951,0.49288166,0.4788281,0.43736935,0.4049897,0.37842375,0.35771725,0.34978256,0.35017928,0.38481727,0.41786832,0.4876936,0.5229572,0.5900206,0.6202182,0.6310979,0.6627909,0.676936,0.6842603,0.69173723,0.69288164,0.65772486,0.64359504,0.58988327,0.55761045,0.51346606,0.5047532,0.4773785,0.4655375,0.46343175,0.49663538,0.52928966,0.59578854,0.62813765,0.6698863,0.7028763,0.7102312,0.7333944,0.74396884,0.70197606,0.66793317,0.6174258,0.60866714,0.5773709,0.5648585,0.55960935,0.55651176,0.59581906,0.6326238,0.70769817,0.7445182,0.8124819,0.8433509,0.8542306,0.8847944,0.8988174,0.9122149,0.91783017,0.88084227,0.8424506,0.7985656,4

0.011841001,0.02059968,0.050614174,0.055924315,0.086823836,0.09423972,0.14767681,0.18066682,0.23085374,0.26474404,0.30988023,0.3444419,0.36386663,0.39815366,0.41257343,0.4265812,0.43002975,0.4331426,0.43353933,0.4334783,0.43141833,0.42795452,0.39729914,0.3862211,0.3575494,0.3432517,0.32544443,0.32014954,0.316701,0.3141375,0.31412223,0.3184558,0.35014877,0.39958802,0.4304875,0.44486153,0.51050586,0.5215076,0.5620203,0.58191806,0.5953002,0.6038148,0.6044099,0.60442513,0.60146487,0.5925231,0.54825664,0.5163958,0.49602503,0.48722056,0.47699702,0.47356373,0.47409782,0.4779736,0.54274815,0.56247807,0.6156558,0.6478218,0.6588388,0.691371,0.7291371,0.74450296,0.7533532,0.76066226,0.7662165

0.027634088,0.05850309,0.06366064,0.09494163,0.10591287,0.14050508,0.20494393,0.23930724,0.31412223,0.35428396,0.39678034,0.4843061,0.5270466,0.6080568,0.6451667,0.71329826,0.727306,0.7645991,0.8033875,0.8052033,0.8122988,0.82105744,0.83077747,0.8319829,0.8291142,0.7917754,0.71201646,0.6728618,0.63263905,0.55846494,0.5253986,0.48798352,0.4526894,0.44367132,0.44130617,0.44029906,0.44103152,0.4448768,0.5087663,0.5388266,0.5874418,0.6178988,0.6552987,0.66265357,0.6820478,0.6952926,0.73266196,0.7365683,0.73812467,0.7377432,0.7338369,0.6657969,0.63189137,0.59867245,0.5348287,0.51497674,0.46831465,0.4371252,0.4165255,0.4056611,0.3967956,0.38959333,0.38307774,0.38533607,0.4494545,0.48343635,0.5190814,0.5911345,0.62430763,0.6701305,0.7042649,0.7438773,0.75388724,0.7914092,0.79342335,0.7920348,0.7859464,0.71352714,0.6791791,0.60895705,0.5754482,0.5434806,0.49317157,0.4870527,0.46491188,0.45975432,0.4566415,0.47855344,0.5146868,0.5493706,0.61751735,0.65009534,0.69217974,0.7257191,0.7542992,0.76299685,0.77022964,0.7820859,0.81721216,0.81561,0.8094606,0.7385672,0.7032273,0.6683299,0.59890133,0.565774,0.5191577,0.4845655,0.44344243,0.43901733,0.4294957,0.42764935,0.4316167,0.4673533,0.5401236,0.5771115,0.65085834,0.6834211,0.7357137,0.7497215,0.76963454,0.79479665,0.82537574,0.8313878,0.8415198,0.8438697,0.8446479,0.84174865,0.8093843,0.7371786,0.70102996,0.62584877,0.5875486,0.5229572,0.49166095,0.48719004,0.4784924,0.47743952,0.47858396,0.48946366,0.56304264,0.59851986,0.67006946,0.7008926,0.7153582,0.75774777,0.7902342,0.80180055,0.8119173,0.81738,0.816907,0.78129244,0.7459373,0.67187,0.63466847,0.5631952,0.5432517,0.52327764,0.49787137,0.49050125,0.49106583,0.53231096,0.56890213,0.6404822,0.6733196,0.70666057,0.75916684,0.7901732,0.81477076,0.8290837,0.8602121,0.8715648,0.8817121,0.8868696,0.88688487

0.010788129,0.021133745,0.03073167,0.041275654,0.0780499,0.08900587,0.119829096,0.15846494,0.16588083,0.20569162,0.23599604,0.24039063,0.25259784,0.26019683,0.24722667,0.23640803,0.21864653,0.20988785,0.1963531,0.18660258,0.22992294,0.23730831,0.27530327,0.2948806,0.31113145,0.3228809,0.33060196,0.32948807,0.29784083,0.2869459,0.26718548,0.2584573,0.23979554,0.22906844,0.23022813,0.24863051,0.27954528,0.2992752,0.31340504,0.322179,0.3347982,0.34187838,0.33548486,0.3243,0.2960403,0.2849775,0.26530862,0.25813687,0.24902724,0.2527199,0.2872053,0.33939117,0.374197,0.4064088,0.44766918,0.4804303,0.49535364,0.50063324

0.004333562,0.0064393072,0.0102693215,0.031906616,0.051819637,0.0713817,0.12217899,0.13316548,0.1996643,0.22992294,0.2845655,0.31746396,0.36263067,0.4,0.4144808,0.45937285,0.49124897,0.5113451,0.5304494,0.53891814,0.53832304,0.537087,0.5299306,0.52648205,0.5184253,0.5178912,0.5183032,0.5195392,0.53168535,0.5631647,0.58113986,0.59206533,0.60218203,0.60482186,0.6064698,0.6076448,0.6074617,0.6037232,0.5925689,0.554284,0.5398642,0.525246,0.5199664,0.5135729,0.513344,0.51482415,0.5183185,0.5604486,0.5648585,0.5869688,0.5913634,0.6000763,0.6011902,0.6016785,0.6010834,0.5947356,0.5836881,0.5605096,0.55425346,0.5473869,0.54691386,0.5472038,0.54920274,0.58834213,0.59700924,0.62717634,0.6459754,0.65329975,0.6597391,0.66137177,0.6624247,0.66208893,0.6555276,0.62121004,0.60125124,0.5547265,0.52382696,0.5193561,0.5053788,0.4995041,0.4983749,0.49904633,0.5030137,0.53879607,0.6045167,0.6399481,0.6736553,0.71201646,0.72256047,0.72700083,0.72867936,0.7276112,0.70800334,0.6641947

0.014999619,0.04556344,0.08180362,0.11219959,0.12091249,0.13142596,0.12704661,0.11798276,0.12356756,0.15669489,0.19929808,0.20540169,0.23111315,0.24170291,0.23189135,0.20645456,0.19528496,0.17544824,0.1680476,0.15571831,0.14998093,0.15512322,0.18706036,0.22983138,0.23590448,0.24991226,0.2635233,0.2711986,0.26976424,0.25754178,0.23782712,0.20485237,0.19383535,0.17613488,0.16876478,0.15725948,0.14984359,0.15565728,0.19142443,0.20544747,0.22165255,0.23076218,0.2274052,0.21853971,0.19659723,0.18574807,0.17694362,0.16087587,0.15344472,0.14721905,0.1359121,0.12904555,0.18272679,0.20250247,0.24068055,0.27087817,0.28230718,0.28561836

0.07460136,0.11270314,0.15507744,0.20071718,0.24881361,0.29779506,0.34579995,0.43192187,0.46823835,0.498558,0.50386816,0.5102007,0.50893414,0.4708934,0.4234226,0.4093843,0.36867324,0.3377737,0.32175174,0.31287098,0.30414283,0.29884794,0.35939574,0.393637,0.44501412,0.47649348,0.4825513,0.49221027,0.48473334,0.4514992,0.40436408,0.39327076,0.35989928,0.32007325,0.31403068,0.283711,0.26935226,0.24724193,0.23834592,0.22964828,0.21832609,0.24968338,0.28447396,0.3540093,0.38710612,0.4012665,0.43256274,0.44351873,0.43903258,0.40892652,0.36992446,0.3264515,0.31226063,0.28398564,0.27302969,0.25288776,0.24199283,0.23327993,0.2186923,0.20801099,0.2103151,0.24605173,0.32312503,0.3622034,0.40016785,0.46674296,0.4756695,0.50438696,0.51299304,0.5134966,0.51209277,0.5082475,0.47635615,0.42914474,0.4180972,0.37737086,0.34584573,0.32964066,0.3153582,0.3045243,0.28834975,0.2777142,0.27284658,0.27734798,0.35489434,0.39464408,0.43327993,0.5038529,0.5232624,0.5430991,0.55440605,0.5592126,0.5596704,0.5583429,0.5176013,0.4705272,0.43910888,0.39514762,0.38109407,0.3473106,0.3328603,0.32172123,0.2966659,0.2797589,0.27772945,0.3420157,0.37686732,0.41063553,0.45162126,0.4660258,0.47217518,0.47679865,0.43599603,0.42178988,0.3772488,0.34476236,0.3071641,0.28253606,0.2715343,0.2641184,0.2567788,0.24614328,0.24216068,0.24200809,0.25717556,0.2980087,0.37941557,0.41690698,0.44802013,0.4532082,0.46030366

0.060273137,0.0957351,0.1321889,0.17143512,0.21385519,0.25940338,0.3563897,0.40674448,0.45711452,0.5571527,0.6069276,0.7006332,0.7436942,0.81898224,0.84962237,0.876524,0.88165104,0.8847028,0.88633555,0.8855421,0.8348669,0.79157704,0.703212,0.66033417,0.6184939,0.5383383,0.5000076,0.4290074,0.39662775,0.35187304,0.31839475,0.27563897,0.26947433,0.25001907,0.23016709,0.21927215,0.2033875,0.19725338,0.18738079,0.17692836,0.17483787,0.17288472,0.1736019,0.21306172,0.25166705,0.34039825,0.39012742,0.44231325,0.54964525,0.6007782,0.692195,0.7308156,0.7689021,0.77759975,0.7848936,0.7853666,0.7848478,0.7427634,0.70350194,0.62858015,0.5907988,0.5168078,0.48107117,0.41452658,0.3835355,0.3692836,0.32462043,0.29222554,0.25468832,0.2501564,0.22824445,0.21733426,0.20862135,0.1941558,0.18883039,0.18028535,0.1772488,0.17355612,0.17286946,0.17347983,0.17651637,0.21127641,0.28708324,0.32893872,0.417319,0.46179903,0.5478294,0.58764017,0.6570077,0.67107654,0.7021134,0.71252,0.71709776,0.722591,0.72330815,0.72115666,0.7013657,0.6670024,0.6211795,0.5841917,0.5156481,0.48372626,0.4338445,0.4017548,0.35735103,0.34851605,0.3176776,0.30148774,0.29280537,0.28357366,0.28126955,0.28015563,0.28152895,0.3123827,0.40383002,0.46089876,0.5850462,0.6469215,0.7629511,0.81353474,0.8918288,0.9006333,0.90960556,0.91186386

0.23938353,0.33206683,0.43219653,0.5329366,0.71847105,0.7982757,0.9295186,0.96475166,0.97094685,0.9368582,0.8958114,0.81513697,0.7720455,0.68889904,0.6504769,0.5790036,0.5465934,0.5269093,0.4892195,0.46987107,0.46567482,0.5249866,0.60042727,0.78884566,0.89228654,0.94613564,0.955108,0.9629511,0.9678798,0.9698787,0.9732204,0.9782711,0.9327077,0.8246128,0.7686885,0.66883343,0.6281224,0.5926604,0.58793014,0.5890593,0.7134661,0.80047303,0.9460441,0.95503163,0.95951784,0.96552986,0.96781874,0.9715877,0.9731594,0.9758297,0.97703516,0.97811854,0.98005646,0.94642556,0.903151,0.81101704,0.7671931,0.68346685,0.6461738,0.6045777,0.58467996,0.5828336,0.5878233,0.64473945,0.7962768,0.8781262,0.9695277,0.97131306,0.9729,0.97560084,0.9768063,0.9789426,0.9798886,0.9807889,0.970779,0.86996263,0.8194705,0.7196765,0.67328906,0.58811325,0.54963,0.4828107,0.47673762,0.4693675,0.46472877,0.4650492,0.5211566,0.5778744,0.6950637,0.7475242,0.8339818,0.86715496,0.87397575,0.87467766,0.87402153,0.8696117,0.8046998,0.76852065,0.7310292,0.65423054,0.6149233,0.53905547,0.502266,0.43356985,0.40225834,0.35252917,0.34718853,0.33765164,0.33749905,0.41338217,0.4631571,0.5786984,0.63945985,0.6962234,0.79139394,0.8260471,0.84901196,0.85243,0.8055695,0.76708627,0.7273365,0.6458686,0.60546273,0.52741283,0.49008927,0.4195926,0.38796064,0.33717862,0.32018006,0.3161822,0.36450753,0.41919586,0.546746,0.60976577,0.6690013,0.75236136,0.7633936,0.7725185,0.76102847,0.6876936,0.6522164,0.61629665,0.54404515,0.5078355,0.43717098,0.40257877,0.33801785,0.32393378,0.29217976,0.2781567,0.356878,0.40805677,0.5208362,0.573785,0.62374306,0.69835967,0.7072862,0.7133288,0.7087663,0.67292285,0.60694283,0.5754177,0.5121996,0.48096436,0.4180972,0.3983215,0.3445487,0.31374076,0.30643168,0.2861219,0.3222095,0.42644387,0.4826276,0.5766232,0.6108949,0.61533535,0.618616

0.0018463416,0.0029755093,0.006347753,0.01075761,0.041123062,0.055344474,0.09202716,0.123277634,0.16165408,0.16775769,0.18721294,0.21020828,0.21915007,0.23396659,0.24122988,0.25181964,0.25575647,0.26179904,0.26416418,0.26604104,0.2697032,0.27113757,0.27162585,0.27226672,0.27248037,0.2725414,0.2723888,0.27203783,0.27055773,0.26929122,0.26582742,0.2637522,0.26062408,0.25992218,0.25978485,0.26135653,0.2640116,0.30058748,0.3067216,0.33525598,0.346334,0.37172502,0.37898833,0.38794538,0.39525443,0.40364692,0.4079652,0.4078584

0.016983291,0.04742504,0.054581523,0.08654917,0.09739833,0.13051042,0.14163424,0.18348974,0.21457237,0.226215,0.23237965,0.24196231,0.23701839,0.22192721,0.2108339,0.19304189,0.18431373,0.17715724,0.16504158,0.15991455,0.15085068,0.1487602,0.14856184,0.14882123,0.1525597,0.15988404,0.19449149,0.2033112,0.22453651,0.2306096,0.24057373,0.24196231,0.24307622,0.24307622,0.24274053,0.237644,0.22597085,0.20825513,0.19942015,0.17969024,0.17441063,0.17454796,0.18318456,0.21870756,0.24992752,0.29794767,0.3088426,0.3440299,0.35025558,0.36214235,0.36508736,0.36766613,0.3678645,0.36733043

0.0055085067,0.007110704,0.01045243,0.019134814,0.04919509,0.054489966,0.08499275,0.12130922,0.12646678,0.15165941,0.16279851,0.18239109,0.18959334,0.20082399,0.20523384,0.20914015,0.21509117,0.21686122,0.21858549,0.21884489,0.21812771,0.21672389,0.21408407,0.20004578,0.18902877,0.16932936,0.14445716,0.13723965,0.12584116,0.11622797,0.10933089,0.1031815,0.102235444,0.10156405,0.101762414,0.104097046,0.10759136,0.11633478,0.14166476,0.15265125,0.1704738,0.17781338,0.19133288,0.19652094,0.20047303,0.20630197,0.20791943,0.20946059,0.20975052,0.20935377,0.2086061,0.19497979,0.1775845,0.16882582,0.15284963,0.14566262,0.1395285,0.12994583,0.123842224,0.12617685,0.16441596,0.16966507,0.19514763,0.20383002,0.21115434,0.22183566,0.22565041,0.22980087,0.23065537,0.23083848

0.0071412222,0.011047532,0.019958802,0.057846952,0.091004804,0.14525063,0.17860685,0.25009537,0.2902266,0.33443198,0.4314641,0.48535895,0.60120547,0.6606546,0.7832609,0.84217596,0.94970626,0.956878,0.9607843,0.9703059,0.9735103,0.97615016,0.9772793,0.9802548,0.9869841,0.98840314,0.9900206,0.99134815,0.99194324,0.9922179,0.9927367,0.99298084,0.99324024,0.9887083,0.9022812,0.8593118,0.8168002,0.7358663,0.6962539,0.6221408,0.589456,0.53670555,0.52257574,0.48116273,0.4497139,0.41539636,0.4042115,0.38583964,0.3797055,0.37198442,0.3717708,0.38719767,0.43254748,0.54326695,0.60704964,0.7424735,0.8104066,0.9381552,0.96906996,0.9708858,0.9739376,0.97529566,0.97763026,0.9786832,0.98056,0.98139924,0.98220795,0.98364234,0.9843137,0.9855344,0.986099,0.98713666,0.98762494,0.9885252,0.9889372,0.9893339,0.9900816,0.9904326,0.99108875,0.9911498,0.94251925,0.8971695,0.8078279,0.76617074,0.72713816,0.6579385,0.63843745,0.608423,0.59432364,0.5855497,0.57886624,0.5781796,0.5814145,0.61991304,0.71020067,0.762417,0.87280077,0.92813003,0.964065,0.9703975,0.97349507,0.97833216,0.98023957,0.98109406,0.9819028,0.9833982,0.98408484,0.9853208,0.98590064,0.9869688,0.9874571,0.9892271,0.9900053,0.9903563,0.99101245,0.9913329,0.98496985,0.94151217,0.85642785,0.8160525,0.7775082,0.7087205,0.68871593,0.64824903,0.6180514,0.60704964,0.6055085,0.60663766,0.6265507,0.7165484,0.7688258,0.88058287,0.9375601,0.9706111,0.97224385,0.97369343,0.9750668,0.98039216,0.9820401,0.98278785,0.98419166,0.98481727,0.98541236,0.98654157,0.9870451,0.9880064,0.9884489,0.98928815,0.9896849,0.99006635,0.99041736,0.9715267,0.89637595,0.86135656,0.8281529,0.78335243,0.75074387,0.7278706,0.7159075,0.7442588,0.7799344,0.86480504,0.9121233,0.96003664,0.9631037,0.97164875,0.9746243,0.97589076,0.9781796,0.97917145,0.980972,0.98179597,0.9832914,0.98397803,0.98463416,0.98582435,0.986389,0.9874113,0.98788434,0.9883421,0.9578393,0.8858625,0.8494392,0.7809262,0.7493553,0.7030747,0.6941482,0.67420465,0.65803003,0.6485847,0.6461585,0.64864576,0.68206304,0.75985354,0.80531013,0.8525826,0.95135427,0.9574273,0.9642634,0.97055006,0.9762417,0.97737086,0.9794156,0.9803464,0.9812009,0.98275733,0.98416114,0.98478675,0.98538184,0.98651105,0.98702985,0.98799115,0.98843366,0.9892729,0.9904021,0.9882353,0.95413136,0.8895247,0.86965746,0.8225681,0.7903563,0.78306246,0.74819565,0.7392996,0.7252766,0.7195239,0.7188983,0.7550011,0.7938811,0.83680475,0.930251,0.9651179,0.9694667,0.971252,0.97891206,0.9798581,0.9815976,0.98239106,0.98382545,0.98449683,0.9857023,0.98626685,0.9867857,0.98777753,0.9882353,0.9890745,0.98947126,0.99021894,0.9821927,0.9419547,0.8697948,0.83654535,0.79102767,0.7566644,0.7457389,0.7096666,0.68815136,0.6834211,0.68398565,0.72635996,0.7690242,0.814435,0.9076829,0.95384145,0.9623865,0.97120625,0.975494,0.97671473,0.9788663,0.9798123,0.9815518,0.9823453,0.98377967,0.98445106,0.9850614,0.9862211,0.98673993,0.98773175,0.9881895,0.98902875,0.99017316,0.98970014,0.94114596,0.9045243,0.8372015,0.8063935,0.75521475,0.7443046,0.7126726,0.6924086,0.6816052,0.6670787,0.66092926,0.6953231,0.7318837,0.77332723,0.8664378,0.9143053,0.9610437,0.9640345,0.97036695,0.97485316,0.9761044,0.9783169,0.9792935,0.9810788,0.9818875,0.98338294,0.9840696,0.9846952,0.9858854,0.98645,0.9874571,0.9879301,0.9888151,0.9892271,0.9900053,0.9788205,0.9412375,0.8941329,0.8637522,0.8563668,0.8540475,0.8531014,0.8542153,0.8604257,0.93463033,0.9673152,0.9711452,0.9727474,0.9754635,0.9766842,0.97778285,0.97979707,0.9807126,0.98153657,0.98504615,0.9877165,0.9901579,0.9908446,0.99176013,0.9937285,0.9969787,0.99707025,0.99740595,0.99795526,0.9980774,0.9982147,0.9982605,0.99835205,0.9983978,0.9984894,0.99853516,0.99858093,0.99864197,0.9986725

0.010879682,0.02449073,0.034912642,0.040024415,0.051834896,0.060471505,0.059632257,0.06099031,0.07150377,0.081177995,0.07971313,0.08160525,0.09140154,0.101136796,0.09918364,0.089662015,0.09059282,0.10028229,0.09875639,0.088380255,0.09176776,0.10305943,0.11076524
